# Naturally Crosslinked Biocompatible Carbonaceous Liquid Metal Aqueous Ink Printing Wearable Electronics for Multi-Sensing and Energy Harvesting

**DOI:** 10.1007/s40820-024-01362-z

**Published:** 2024-03-11

**Authors:** King Yan Chung, Bingang Xu, Di Tan, Qingjun Yang, Zihua Li, Hong Fu

**Affiliations:** 1 Nanotechnology Center, School of Fashion and Textiles, The Hong Kong Polytechnic University, Hong Kong, 999077 People’s Republic of China; 2 Department of Mathematics and Information Technology, The Education University of Hong Kong, Hong Kong, People’s Republic of China

**Keywords:** Biocompatible, Conductive ink, Biopolymer, E-textile, Carbonaceous liquid metal

## Abstract

Achieving flexible electronics with comfort and durability comparable to traditional textiles is one of the ultimate pursuits of smart wearables. Ink printing is desirable for e-textile development using a simple and inexpensive process. However, fabricating high-performance atop textiles with good dispersity, stability, biocompatibility, and wearability for high-resolution, large-scale manufacturing, and practical applications has remained challenging. Here, water-based multi-walled carbon nanotubes (MWCNTs)-decorated liquid metal (LM) inks are proposed with carbonaceous gallium–indium micro-nanostructure. With the assistance of biopolymers, the sodium alginate-encapsulated LM droplets contain high carboxyl groups which non-covalently crosslink with silk sericin-mediated MWCNTs. E-textile can be prepared subsequently via printing technique and natural waterproof triboelectric coating, enabling good flexibility, hydrophilicity, breathability, wearability, biocompatibility, conductivity, stability, and excellent versatility, without any artificial chemicals. The obtained e-textile can be used in various applications with designable patterns and circuits. Multi-sensing applications of recognizing complex human motions, breathing, phonation, and pressure distribution are demonstrated with repeatable and reliable signals. Self-powered and energy-harvesting capabilities are also presented by driving electronic devices and lighting LEDs. As proof of concept, this work provides new opportunities in a scalable and sustainable way to develop novel wearable electronics and smart clothing for future commercial applications.

**Supplementary Information:**

The online version contains supplementary material available at 10.1007/s40820-024-01362-z.

## Highlights

- Naturally crosslinked carbonaceous liquid metal aqueous printable ink mediated by biopolymers.
- E-textile with conductivity, stability, wearability, and aesthetic characteristics.
- Multi-applications in health monitoring, pressure sensing, and energy harvesting.

**Supplementary Information:**

The online version contains supplementary material available at 10.1007/s40820-024-01362-z.

## Graphical abstract

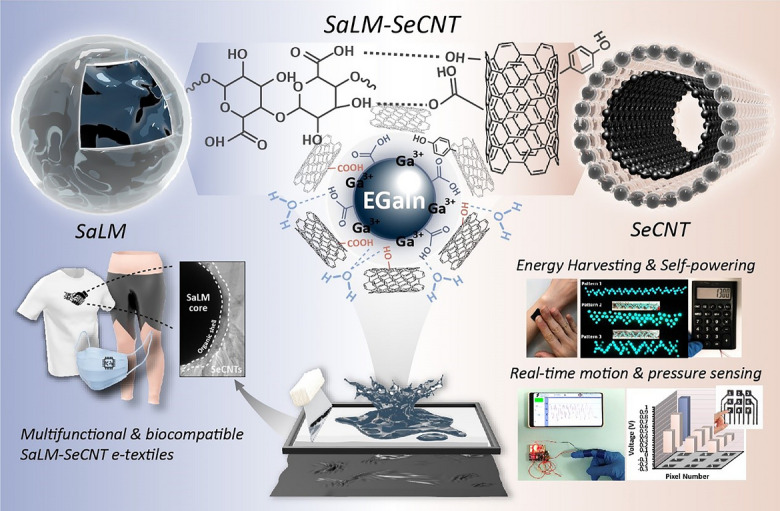

## Introduction

Research on wearable electronics has attracted increasing attention in recent days owing to their great potential in health and motion monitoring [1–3], human–machine interfaces [4–6], and virtual/ augmented reality [7]. To possess the function of electronics and superior wearing comfort, e-textiles show ideal features such as outstanding mechanical properties, good flexibility and softness, conformal contact with skin, and excellent wearing comfort [8–11]. Particularly, ink-based techniques have been realized rapidly in fabricating versatile advanced smart clothing devices, such as flexible circuits [12, 13], sensors [14–17], and self-powered and energy devices [9, 18–23], by introducing conductive inks on flexible substrates that offer simplicity, low-cost, scalability, and a remarkable low wastage [24, 25]. The selection of conductive inks shows an important issue dominating the balance between conductivity, processability, stability, adhesiveness and other issues such as cost. Biocompatibility has also become a desirable property for conductive inks since the intensive development of wearable electronics and bioelectronics devices [26]. Water is the most environmentally friendly solvent which has a low-cost, nonflammable nature, and outstanding biocompatibility, motivating the search for more efficient procedures to produce water-based conductive inks [24]. The development of green solvents usually survives with poor dispersion and adhesiveness in most conductive compounds that binders or surfactants are required to induce the homogeneity of high-quality conductive ink. However, the common use of polymeric binders is not affinity with water-based solvents which causes a dilemma in fabricating biocompatible aqueous ink. Improving the stability and robustness of the interfacial interaction between conductive components and water-based solvents are extremely critical to exert for “true” natural conductive ink. Moreover, adhesiveness between conductive ink and textile survives from the instable dispersion because of the porous, rough and anisotropic structure. Therefore, it is highly essential to develop new smart electronic textiles (e-textiles) through a facile and efficient manufacturing technique that can not only integrate the functionality of electronics and the comfort of textiles but also enable good eco-friendly, biocompatibility and adhesive features for future multi-function commercial applications of a variety of wearable electronics.

Notably, gallium-based liquid metals (LMs) have been widely considered, given their superior electrical conductivity, low melting point, high thermal conductivity, and inherent liquid fluidity [27, 28]. In contrast to solid fillers, LM nanodroplets (LMPs) can deform with stretchable substrates, suggesting its superiority in forming integral materials to obtain multifunctional flexible devices [29–32]. In most cases, the contribution of LMPs via sonication was susceptible to oxidation and aggregation which led to non-uniform dispersion and reduced functionality, especially for water-based suspension [33]. To deal with the insufficient colloidal stability, endeavors have been taken through surface modification [34, 35] but still lack sufficiency to fulfill high environmental and biological safety. Recently, a study has been proposed using alginate which could encapsulate EGaIn nanodroplets into microgels of marine polysaccharides and maintain good stability [36]. Although this work has benefited the development of aqueous LMPs ink, the electrical and thermal conductivity was weakened inevitably due to the intrinsic insulation of these surfactants. Instead of injecting additive materials, an alternative work reported an interface interaction structure with the assistance of carbon nanotubes (CNTs) to improve the LM dispersion in water [37]. Uniform dispersion and stability were obtained but were restricted to the homogeneity function because of the ease of cracking and limited stretchability. Moreover, critical sintering was required in LM-based research to recover the electric conductivity which largely hinders the ease of the manufacturing process and wide applications, specifically to smart textiles.

Taking consideration of hybrid composition, carbon nanotubes (CNTs) have been regarded as a promising active material because of their unique electrical properties, mechanical robustness, better biocompatibility, and high stability [13, 38]. Previously, various approaches were adopted to address the problem of self-aggregation [39, 40] and cytotoxic [41, 42], as well as to improve the development of green-based CNTs inks. Lately, sericin as a bio-stabilizer in modifying the surface of CNT was revealed [43, 44], which could promote strong *π*–*π* interactions with the surface of CNTs and the high amphipathic nature reduced the surface energy, resulting from highly stabilized CNTs water dispersion. The fabricated ink presented uniform dispersion, good hydrophilicity, and biocompatibility, endowing it with large-scale applications in printed electronics. In this regard, the great potential of supramolecular adhesion can be recognized among biopolymers owing to the non-covalent bonds, such as DNA and RNA recognition, protein folding, and cell adhesion in life processes [45]. Alginate is one of the most abundant biopolymers in the ocean and has plenty of carboxyl groups in its uronic units, while sericin is built up by amino acid residues linked with peptide bonds and serves as an adhesive “glue” to bind fibers together in silk cocoons [46]. Based on this principle, it is expected that strong natural and stable interaction could be achieved by the decoration of LMPs with rich functional groups (acting as adopters) and MWCNTs with rich adhesive groups (acting as promoters) in a water solvent to a form an egg-shell structure, endowing the development of biocompatible printable conductive ink.

Here, this study proposes a naturally crosslinked biocompatible carbonaceous liquid metal aqueous ink and demonstrates versatile applications in wearable electronics via printing techniques for multi-sensing activities and energy harvesting. The addition of rich biopolymers, sodium alginate (Sa), and silk sericin (Se), enables the uniform and stable dispersion of LMs-MWCNTs ink without any artificial chemicals, ensuring its good biocompatibility and potential in green electronics. A strong chemical interaction is successfully formed through hydrogen bonds between the hydrophilic-rich groups of SeCNTs and the rich carboxyl groups of the SaLMs, which leads to the stabilization effect. The fabricated composite shows high dispersity and re-dissolvable ability in water, and consequently, the highly homogeneous conductive ink can be applied to various flexible substrates (e.g., textiles) via printing techniques with high-resolution and aesthetic features. The patterned e-textiles exhibit electrical conductivity, stretchability, good wearability, and dynamic stability which endow its versatile real-time applications in human health monitoring, pressure sensing, and energy harvesting. Hence, the development of carbonaceous liquid metal aqueous ink sheds a stable core–shell architecture, new ideas in the development of green-based conductive ink, and expends the exiting e-textile to the form of multifunctional applications with decorative feature via cost-effective way.

## Experimental Section

### Materials

Liquid metal consisting of Gallium metal (bulks, > 99.9%) and indium (beads, > 99.9%) was obtained from Huaxia Reagent Co., Ltd. (Chengdu, China). Sodium alginate (weight-average molecular weight, *M*_w_ ≈ 200,000–300,000) was purchased from Shanghai Aladdin Biochemical Technology Co., Ltd. Multi-wall carbon nanotubes (MWCNT) were produced by Nanjing XFNANO Materials Tech Co., Ltd. Sericin (SS) power content 10 wt% (per hundred rubbers) was obtained from Macklin Inc. Water polyurethane (WPU, solid content: 40%) was purchased from Shanghai McLean Biochemical Co., Ltd. Hydroxyethyl cellulose (HEC, 80–125 mPa s, 25 °C) was obtained from Shanghai Aladdin Biochemical Technology Co., Ltd.

### Preparation of Sa-coated LM Nanodroplets (SaLMs)

Typically, bulk EGaIn (150 mg) was added into an aqueous solution (15 mL) of sodium alginate (0.3 wt%). The mixture was exposed to a probe sonicator (BILON92-II; power of 750 W with 30% amplitude) in an ice-water bath for 30 min to achieve uniform EGaIn droplets. The resultant suspension was washed with water more than three times by centrifugation (5000 rpm, 10 min).

### Preparation of SeCNT-Wrapped SaLM Nanodroplets (SaLM-SeCNTs)

The sericin aqueous solution was obtained by stirring sericin power (0.05 g) and deionized (DI) water (10 mL) followed by 10 min sonication using a probe sonicator. A certain designed amount of SaLMPs was placed in a beaker, which was filled with an equal amount of the silk sericin-CNT (SeCNT) mixed solution. The mixture was then sonicated properly for 30 min to generate SeCNT-wrapped LMP nanodroplets. Afterward, the hybrid nanodroplets in the obtained suspension were collected by centrifugation at 5000 rpm for 15 min and lyophilization for further use. The different weight ratios of SeCNT-wrapped SaLMP Nanodroplets (ink-0.5, 1, 2, 3) were prepared, respectively.

### Synthesis of SaLM-SeCNT Conductive Ink and Printing Technologies

The obtained SaLM-SeCNT particles were re-dispersed into water solution and stirred properly, in order to achieve a uniform printable ink. Printed SaLM-SeCNT wearable electronics were fabricated by depositing the ink on various substrates (i.e., planar substrates and textiles) using different methods, respectively. (1) Rigid and Flexible Substrates: rollerball pen drawing was used. The ink of commercially available rollerball was replaced with the SaLM-SeCNT dispersion. Various substrates were used, including copy paper, weigh paper, copper paper, glass slide, glove, WPU film, and PDMS film. Particularly, glass slide and PDMS film were treated with air plasma before depositing to increase the affinity to water. (2) Textile substrates: the weft-knitted polyester textile was selected as the substrate. A patterned screen mask was prepared by the Riso screen maker. The ink was then squeezed onto the textile surface properly. To achieve lower resistance, the circuits were printed several times in the above methods.

### Fabrication of SaLM-SeCNT Triboelectric Fabric

4 mL WPU and 10 mL HEC (3 wt%) was added into 6 mL DI water (in a 50 mL beaker) and kept stirring. After magnetic stirring (1000 r min^−1^) for 1 h, the HEC-WPU mixture was successfully prepared. Finally, the HEC–WPU mixtures were uniformly integrated into the prepared conductive fabric by a blade-coating method for obtaining the triboelectric fabric.

### Characterization

Morphology, microstructure, and elemental analysis of SaLM-SeCNT nanodroplet were assessed through Transmission Electron Microscope (JEOL JEM-2010), Raman Spectrometer (Bayspec Nomadic III Laser Raman Confocal Microscope), Fourier Transform Infrared Spectrometer (FTIR) (PerkinElmer FTIR Spectrum 100 + Autoimage IR Microscope), and Thermogravimetric analyzer (TGA) (Perkinelmer TGA 4000 System 100–240 V/50–60 Hz). SEM was performed on a “Hitachi” Model TM-3000 Tabletop Scanning Electron Microscope. To investigate the mechanical properties and electrical resistance of the printed SaLM-SeCNT textile, real-time resistance was recorded using a Keithley-2400 connecting with a digital multimeter. Testing the wearables was carried out with the assistance of one human participant volunteer (one author of this article), and informed written consent was obtained from the participants. The water contact angle was recorded using SDC-350 contact angle measurement equipment. The washing test was processed by immersing the printed e-textile into the water with washing powder and properly kneading, and then drying the printed e-textile with a hairdryer after removing all the washing powder. The air permeability was measured using a KES Air permeability Tester. The abrasion test was performed by Martindale Abrasion Test (M235/8 8 Head Martindale Abrasion and Pilling Tester). Concerning electric output measurement of the SaLM-SeCNT e-textile, a Keyboard Life Tester (ZXA03) was utilized to operate the periodic contact-separation action with different forces and frequencies. The open-circuit voltage (*V*_OC_), short-circuit current (*I*_SC_), and short-circuit charge transfer (*Q*_SC_) were measured by an electrometer (Keithley 6514 system). The force signal was monitored by DAQ (Dewetron, Dewe-2600 DAQ system).

## Results and Discussion

### Design Principle and Fabrication of the SaLM-SeCNT Conductive Ink

**Fig. 1:**
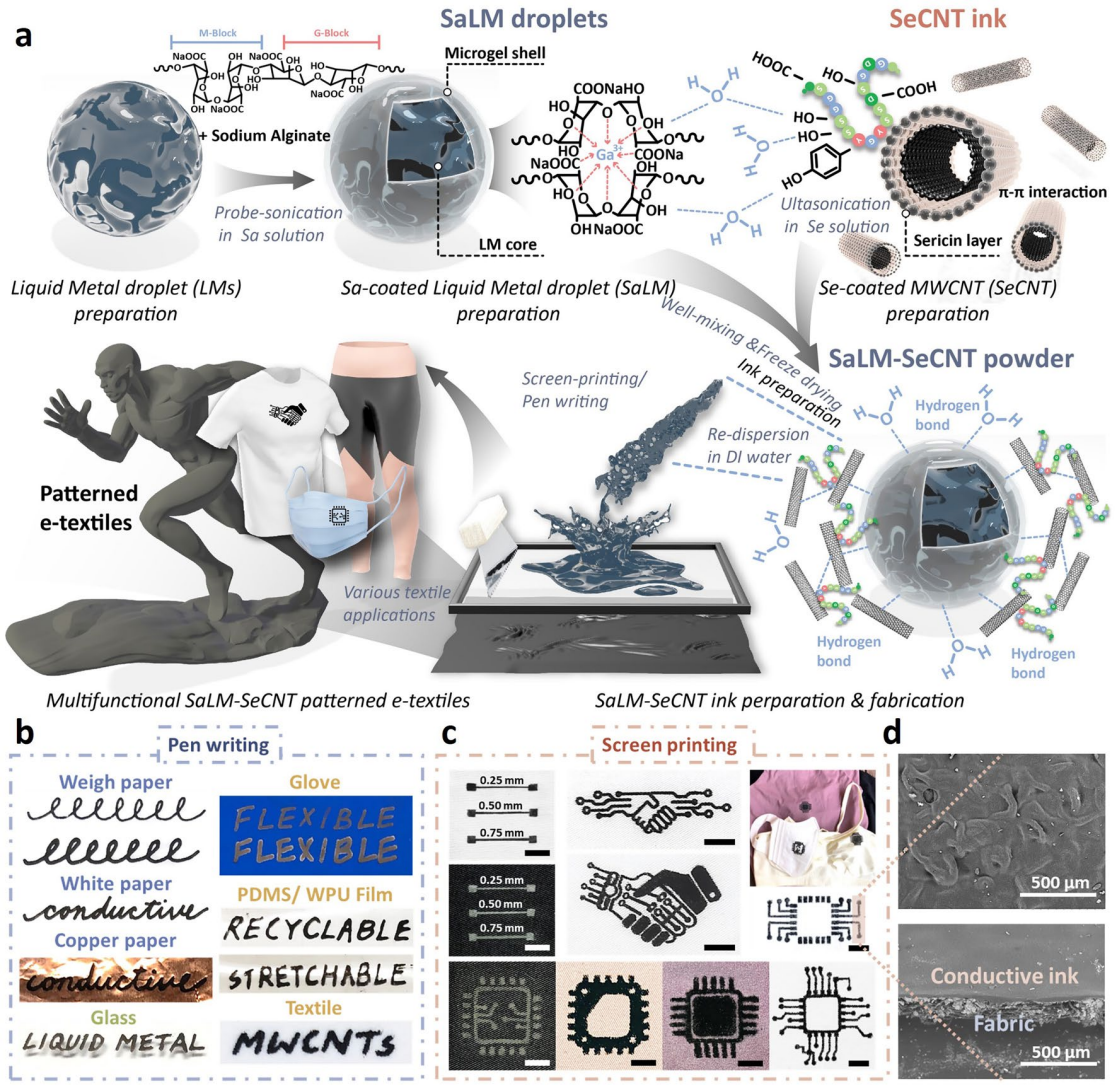
**a** Synthetic roadmap to convert bulk LM to SeLM-SeCNT nanodroplets and the fabrication of water-based SeLM-SeCNT conductive ink for multiple applications. **b** Hand-written traces with a ballpoint pen filled with the SeLM-SeCNT inks. On various flexible substrates. **c** Photograph of printed aesthetic e-textiles with exquisite conductive circuit patterns using screen-printing method on different fabric materials and commercial clothing. **d** SEM image of the printed conductive layer on a textile substrate and the sectional view of the printed e-textile with a resolution of 500 μm

It is a formidable challenge to obtain water-based gallium–indium (EGaIn) and MWCNT-based conductive inks with desirable comprehensive performance, due to their high surface energy and poor wettability. Optimizing the uniformity and stability of conductive ink is proposed by surface modification, in order to induce strong crosslinking networks between LM droplets and MWCNTs and their adherence to different substrates as aqueous ink. To meet the requirements, the design principle of conductive ink should be simple, low-cost, and biocompatible but functional. Figure 1a schematically portrays the fabrication of the water-based conductive ink from bare LM nanodroplets to Sa-coated LM (SaLM) and to SeCNT-wrapped SaLM nanodroplets (SaLM-SeCNT), which demonstrates the ballpoint pen writing and screen-printing process of the water-based SaLM-SCNT conductive ink with the merits of being dispersible, biocompatible, hydrophilic and aesthetic. The synthesis of water-based SaLM-SeCNT ink was based on the top–down approach. Firstly, Sa solution containing EGaIn was subject to probe sonication for 30 min (see the Experimental Section for details), where the ultrasound stimulation disintegrated bulk EGaIn into nano- and micro-droplets. The use of sodium alginate could immediately rupture EGaIn into nanodroplets and obtain a stable opaque slurry. During sonication, the carboxylic groups within the alginate G-segments were chelated with the multivalent cations [47, 48], and the Ga^3+^ produced from oxidizing Ga also interacted with the alginate [49, 50], resulting from ionically crosslinked organic gel shell. Simultaneously, MWCNTs were modified by silk sericin to overcome the strong Van de Waal force and formed SeCNT. The amino acid residues linked with peptide bonds found in silk sericin could regulate strong *π–π* interactions with the surface of CNTs [51]. The coexistence of hydrophilic and hydrophobic groups within sericin makes it amphipathic and therefore endows it with the ability to reduce the surface energy and stabilize the dispersion of CNTs in water. Taking advantage of both surface-modified LMs and MWCNTs, strong crosslinking networks could be probed between the Sa-coated LM droplets and SeCNT to generate a highly uniform dispersion by proper mixing and sonication. The SaLM-SeCNT powders were prepared with colloidal, chemical stability, and biocompatibility. As each SaLM core was coated with SeCNTs and formed a compatible network, no additional sintering operations were required in the whole process. The SaLM-SeCNT ink showed great potential in composites for the writing/patterning and printing process. Figure 1b, c schematically demonstrates a writing method with the inks using a ballpoint pen and a screen-printing method, respectively. Various substrates were employed ranging from rigid, and flexible to stretchable (e.g., paper, glass, glove, PDMS/TPU film, and textile), suggesting considerable adhesive, stability, and multi-applicability. In particular, well-printed aesthetic patterns were presented on textile substrates in which the conductive ink could achieve a line of 0.25 mm width or a resolution of 300 ppi, even under a high resolution, indicating the significant dispersion quality of the ink (Figs. 1c and S1, left). As shown in Fig. 1d, a dense interconnected SaLM-SeCNT thin film can be seen on the fabric surface and in the cross-sectional view with a high resolution of ≈500 μm which accesses the eligibility for printing on various fabrics with different textile constructions (e.g., woven, knit, lycra) and materials (e.g., PET, cotton, and wool). More importantly, the printed e-textiles offer significant conductivity (Fig. S1, right), softness, and flexibility including stretching, bending, twisting, and folding, confirming the capability for a wide range of applications, especially for smart and functional clothing.

### Characterization of the Structure and Basic Properties of the SaLM-SeCNT Ink

**Fig. 2:**
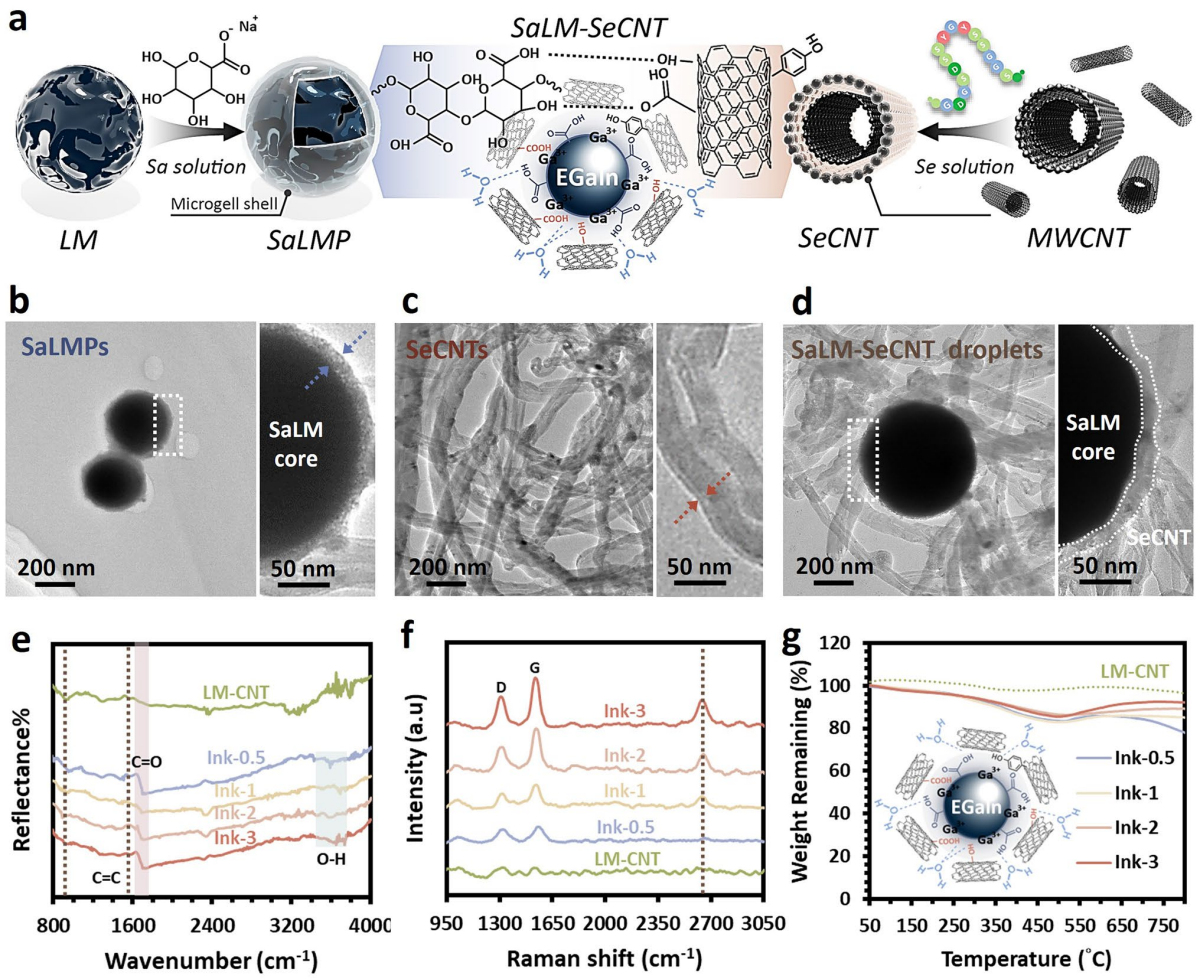
Characterization of SaLM-SeCNT composite. **a** Schematic illustration describing the fabrication procedure and schematic representation exhibiting SaLM nanodroplet decorated with carbonaceous shell SeCNT. TEM image showing **b** the LM nanodroplet with sodium alginate coating and the magnified image showing an EGaIn droplet coated with Sa microgel, **c** the uniformly dispersed MWCNTs prepared from the Se solution and magnified image showing an MWCNT coated with sericin, and **d** the SaLM-SeCNT nanoparticles. **e** FTIR spectra. **f** Raman spectra and **g** TGA curves of LM-CNT and SaLM-SeCNTs nanodroplets (ink 0.5, 1, 2, 3)

The hypothesis on the mechanisms underlying the structure of SaLM-SeCNT was preliminarily examined by transmission electron microscopy (TEM) images. Starting from bulk LM, it was micronized by powerful sonication in Sa aqueous solution and resulted from Sa-coated LM nanodroplets with polydispersity. The high carboxyl groups on the surface of Sa-coated LM nanodroplets were then combined with sericin-modified MWCNTs aqueous dispersion. The hydrophilic-rich groups of adsorbed sericin induced MWCNTs to form hydrogen bonds which could act as the “carbonaceous web” and form crosslinking networks with the rich carboxyl groups on SaLM nanodroplets (Fig. 2a). The globoid LM nanodroplet is tightly coiled by SeCNTs, suggesting the presence of strong interactions between the surface of the LM nanodroplets and SeCNTs as seen in Fig. 2b–d, validating that SaLM nanodroplet surfaces can be efficaciously encapsulated by SeCNT. The TEM image distinctly showed the core-microgel shell structure of the SaLM nanodroplet which was obtained spontaneously during the sonication (Fig. 2b), and the Sa molecules were able to attach to the oxide layer of the LMs via metal coordination. Synchronously, uniformly dispersed SeCNTs with an interlayer spacing were also confirmed (Fig. 2c), consistent with the previously reported diameter for the walls of the CNT and the adsorbed sericin [52, 53]. To investigate the fact that there is a crosslinking between the SaLM nanodroplets and SeCNTs, a middle layer can be observed in Fig. 2d. Of note, the structural attributes and chemical composition further verified the presence of the hydrogen bond crosslinking networks between SaLM-SeCNT. It was taken by comparing pure LM-CNT to four SaLM-SeCNTs samples with different conductive loading ratios. The former (i.e., pure LM-CNT) was constructed with no addition of Sa and Se whereas the latter four were prepared with the addition of Sa and Se. Aiming to study the effect of SeCNT fillers in generating stable non-covalent bonds, four SaLM-SeCNTs samples were conducted following four different weight ratios of SaLM to SeCNT from 0.5, 1, 2, to 3 and were labeled as Ink-0.5, Ink-1, Ink-2, and Ink-3, respectively. Detailed information is provided in Table S1. The signal of metallic Ga and Ga_2_O_3,_ the carbon bonds (C=C), and carbon–oxygen bond (C=O and O–C=O) in LM-CNTs and SaLM-SeCNTs can be defined by FTIR spectra in Fig. 2e. The characteristic peak attributable to the Ga and Ga_2_O_3_ located at 950 cm^−1^ was decreased significantly upon the increase of SeCNTs. This phenomenon was mainly ascribed to the formation of a complexation reaction between Ga^3+^ ions with carboxyl functional groups on the CNT [36, 54]. The double carbon bond (C=C) located at 1670 was also decreased by increasing the addition of SeCNTs spectra. A new absorption peak corresponding to the carbonyl group (C=O/O–C=O) motif at 1710 cm^−1^ in the SaLM-SeCNT proved the carbonyl group and bonding in the SaLM-SeCNT droplet which was shifted to low-wavenumbers due to the strengthened interaction between sericin and CNT with the increasing content of CNTs. In addition, the stretching vibration peak of O–H also appeared significantly broad and with higher intensity in SaLM-SeCNT which was shifted from 3466 to 3585 cm^−1^. The shift of these peaks attested to hydrogen bonds between SaLM and SeCNT. Additionally, the interface strength between SaLM cores and SeCNT wrappings could be further evaluated by Raman spectrum. The peaks of the D band (*I*_D_) and G band (*I*_G_) for the SeCNT web in the SaLM-SeCNT with different ratios are summarized in Fig. 2f, which reflects the evolution of crosslinking networks to some extent. The *I*_D_ was related to the disorder degree of the system, whereas the G peak was attributed to the carbon atoms of CNTs having a complete hexagonal structure (*sp*^2^ hybridization) [55], which could measure the defect concentration of SeCNTs and determine the interface strength [56]. As presented in Fig. 2f, the *I*_D_/*I*_G_ of LM-CNT and SaLM-SeCNT is shifted from 1339 to 1347 cm^−1^ and 1554 to 1568 cm^−1^, respectively, in which the *I*_D_ to *I*_G_ ratio increases from 0.144 to 0.383, showing an enhancement of > 100%. Moreover, the *D* peak and *G* peak also increased from 110.0 of the Ink-0.5 up to 219.5 of the Ink-3, and 128.4 of the Ink-0.5 up to 345.7 of the Ink-3, respectively. It reveals that more defective structures on SeCNT made it easier to form a strong interface interaction with SaLM, thereby obtaining a better performance on the composites which will be discussed later. Additionally, the content of Sa and Se coating in the SaLM-SeCNT nanodroplet was roughly evaluated by thermogravimetric analysis (TGA). It notes that pure LM-CNT nanodroplets demonstrated relatively stable thermal stability due to the lack of a polymer layer. As for SaLM-SeCNT, a continuous weight loss of 20 wt% is observed till 500 °C, corresponding to the thermal degradation of the Sa and Se layer (Fig. 2g). These results manifest that the interaction of SeCNT on the surface of SaLM cores could be efficiently constructed based on the proposed strategy.

### Characterization of the SaLM-SeCNT Ink as e-Textiles

The biocompatible SaLM-SeCNT aqueous ink used for screen printing was optimized to ensure its ink characteristic and performance as e-textiles. After ink disposition, a facile mechanical stretching technique (i.e., stretching at 50% > 20 times) was adopted to rupture the oxidation shells of the LM droplets and drive their coalescence to achieve metallic conductivity linked with SeCNTs. The robust stretching could lead to a friction process in which each SaLM-SeCNTs coated yarn is rubbed continuously with adjacent yarns inside the fabric structure and thus, ruptures the LM shell to return its conductivity. Two important variables were considered including the conductive fillers and water solvent. The former refers to the weight ratios of SaLM to SeCNT in generating the conductive composite, while the latter refers to the weight ratios of the conductive fillers to solvent in fabricating the ink. Aiming to compare the effect of conductive fillers, four different SaLM-SeCNT samples were formulated by adjusting the proportions of SaLM to SeCNT (i.e., 0.5, 1, 2, and 3), followed by dispersing in a fixed water solution (labeled Ink-0.5, Ink-1, Ink-2, and Ink-3), respectively. For the effect of water content, each as-obtained SaLM-SeCNT with different proportions was used to form ink in which the conductive loading wt% in solution was set as 5, 10, 20, and 40 wt%, respectively. As expected, the conductive ink could be simply generated by re-dispersing and proper stirring of the as-prepared SaLM-SeCNT composites in a water solvent. The good stability, uniformity, and biocompatibility of the ink could be affirmed by a long time of standing without precipitation and the assistance of any artificial additives (Fig. S2). Then, the rheological properties of the SaLM-SeCNT ink were examined by comparing the four as-mentioned inks and different wt% of SaLM-SeCNT in water (e.g., 5%, 10%, 20%, and 30%). It was found that the viscosity of the ink was affected by increasing the amount of SeCNTs because of the high aspect ratio and rich functional groups, which can exhibit abundant linkages in the ink (Fig. S3). Moreover, the viscosity of different wt% of SaLM-SeCNTs in fabricating the printable ink is also studied in Fig. S4 which sophisticates the estimation of its printability based on viscosity. The viscosity was decreased with the increase in shear rate, which is necessary for the continuous flow of printable inks. Furthermore, when the ink-20% was inverted, it could still tightly adhere to the bottle wall, implying its nature ability (Fig. S4, inset). The shear-thinning behavior of SaLM-SeCNT ink was favorable in extruding from the screen mesh onto the target substrate during the squeezing process.

**Fig. 3:**
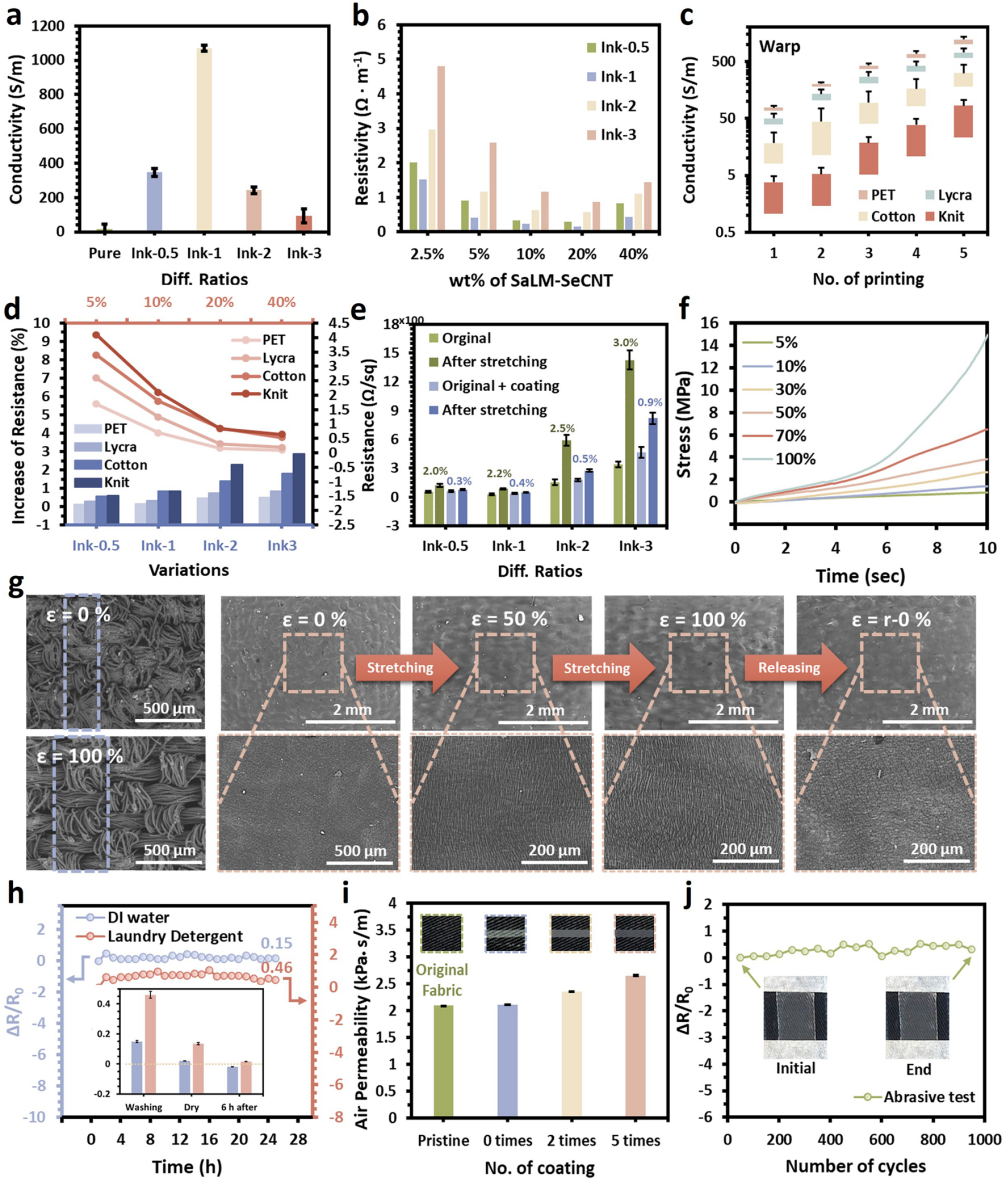
Electromechanical and textile properties of the SaLM-SeCNT conductive ink for printed e-textiles. **a** Conductivity of the SaLM-SeCNT ink with different volume fractions of SaLM-SeCNT. **b** Conductivity of the SaLM-SeCNT ink with different weight ratios in water solution. **c** The effect of No. of printing time and printing directions (warp) on the conductivity of the SaLM-SeCNT ink-printed textiles. **d** The increase (%) of resistance for the printed e-textiles with different textile substrates and SaLM-SeCNT inks (upper *x*-axis: different weight ratios in water; bottom *x*-axis: different fractions of SaLM to SeCNT) after being subject to applied strains. **e** Comparison of resistance changes over multiple stretching cycles with or without WPU–HEC coating. **f** Mechanical properties of the SaLM-SeCNT ink e-textile at different strain%. **g** Surface morphologies and SEM images of the SaLM-SeCNT ink e-textile when stretch to a strain of 0%, 50%, 100%, and restored stage, respectively. **h** Relative resistance of a printed e-textile immersed in DI water and laundry detergent for 24 h. The inset shows the relative resistance after washing and drying. **i** Air permeability of the original textile, after conductive patterning, and with 2 times to 5 times of WPU–HEC coating. **j** Relative resistance change of the initially printed e-textile and the printed e-textile with 1000 abrasions

Regarding electrical conductivity of the SaLM-SeCNT ink, the sheet resistance was first measured without coating on textiles, as shown in Fig. 3a. Compared with pure LM-CNT ink (i.e., without modification of biopolymers), the conductivity of those composites with Sa and Se gives a better conductivity. A rapidly increasing trend could be found between Ink-0.5 and Ink-1 owing to the increase in the SeCNT loadings. A maximum conductivity was received in Ink-1 which was about 50 times higher than that of pure ink. A decreasing trend was then found in Ink-2 and Ink-3, confirming the best interfacial interaction of SaLM and SeCNT in the ratio of Ink-1. Then, different e-textile samples were prepared by screen-printing as-mentioned ink samples and coating them with a protective layer made of water polyurethane (WPU) and hydroxyethyl cellulose (HEC) (WPU–HEC layer) (see the Experimental Section for details). An overall best conductivity and a minimum resistivity of 0.156 Ω m^−1^ were captured in ink-1 with the 20 wt% of SaLM-SeCNT in water, respectively (Fig. 3b). The beneficial conductive effect can belong to (1) the homogenous dispersion of SaLM and SeCNT in the ink; (2) the association of strong crosslinking between SaLM cores and SeCNTs with dense stacking networks. The higher resistivity of printed e-textile using Ink-3 with 2.5 wt% of SaLM-SeCNT is attributed to the less connected conductive networks induced by the lower SaLM loading and viscosity of the ink, as addressed in Fig. 3b. Instead of loading ratios and wt% in DI water, the effect of printing times and printing direction on the conductivity was also studied by printing on diverse textile with different weaving structures, including PET/cotton woven, knit, and lycra. Because of the porous, rough, and anisotropic textile substrate, the conductivity can be affected in which the printing time is due to the capillary process and printing direction is due to the anisotropic structure. Generally, woven and lycra fabrics achieved a lower resistance on average than knitted fabrics (Fig. S5). As depicted in Fig. 3c, there is a fast increase in conductivity when the printing times increases from 1 to 3 layers, and slow down from 4 to 5 layer to reach a maximum range. More importantly, the printing direction along the warp direction of the fabric substantially presented better conductivity, while PET fabric and Lycra gave a higher printed conductivity because of the fabric construction (Figs. 3c and S6).

Additionally, the resistance change of the e-textile with different ratios of SaLM-SeCNT inks was probed and compared before and after being subject to applied strain. Figure 3d indicates that the ink with lower ratios of SaLM to SeCNT (bottom *x*-axis) and higher wt% in DI water (upper *x*-axis) engendered relatively better conductive stability and offered a smaller increase in resistance after deformation. The proper proportion ratios of the SaLM to SeCNT controlled conductivity with effective pathways; water content promoted strong crosslinking to the SaLM-SeCNT particles. Among the selected fabric substrates, PET woven fabric promoted better resistance to deformation while knit fabric showed the largest increase (%) in resistance (Fig. S7). Moreover, an overall improvement in stable resistance change was found by coating the WPU–HEC layer to a range of 0.3–0.9%, compared to those without coating (i.e., a range of 2–3%). The results concluded the conductivity and mechanical stretchability of the SaLM-SeCNT ink with a WPU–HEC coating (Fig. 3e). Therefore, in the following sections, all the printed e-textiles were fabricated by Ink-1 with 2 times WPU–HEC coating unless otherwise stated.

Furthermore, the printed conductive pattern caused insignificant changes to the flexible nature of textiles, including thickness, bending, and extension level, ensuring its feasibility for various types of fabrics (Figs. S8–S10). A certain level of stretchability was maintained against the mechanical change in the textile with WPU–HEC coating which was further manifested by the stress–strain curves (Fig. 3f) and the surface morphologies of the original textile at the initial state, stretched state (100% strain), and restored state (Fig. 3g). The elongation of the printed layer was obtained by the heterogeneous properties of the weave structure in textiles. When the textile sheet was stretched, the yarns were stretched slightly and the weave gaps were significantly enlarged in the weft direction (Fig. 3g left). As shown in Fig. 3g, the SaLM-SeCNT conductive ink on the textile can be against a tensile strain of 100% with no obvious breakage on the surface. Although extremely few and small cracks could be seen, they appeared in tidy and regular orders, resulting in continuous resistance change (more information will be stated in the next section). Remarkably, the conductive layer could mostly return to its initial surface with ignorable wrinkles once the applied strain was released. The robustness to stretchability can be explained by (1) the improved architectural structure between the SaLM core and the SeCNTs web in which the microgel shell-wrapped LM cores and the high aspect ratio of SeCNT promoted good crosslinking and strong adhesive in forming hydrogen bond in water and (2) the strong penetration of WPU–HEC coating to improve structural compaction and interconnection between SaLM-SeCNT “mortar” and textile “brick”.

As a kind of wearable electronics, the water resistance, washability, breathability, and wear resistance of the printed e-textile were evaluated. Indeed, the water-based SaLM-SeCNT ink itself could be re-dispersed after drying due to its good hydrophilicity, leading to questionable washability as a wearable device. Therefore, the WPU–HEC coating was important for protecting the conductive ink against water and improving the hydrophilicity. The driving force between the coating and textile was estimated, in which a shadow of conductive ink was kept on the fabric surface after peeling (Fig. S11). The cyclic peeling test in Fig. S12 also confirmed the stability of the ink-textile bonding in which no significant change was found in the conductivity. Figure S13 presents the wettability of the printed e-textiles by measuring the contact angle of the water droplet which becomes stable (> 90.0°) in the hydrophobic condition compared to the e-textile without coating (< 30.0°). Owning to the hydrophobicity, the printed e-textile maintained most of its initial electric conductivity reliably with < 10% change of relative resistance after soaking in DI water and laundry detergent for more than 24 h (Fig. 3h). The washability of the e-textile was characterized by monitoring the resistance of the textile using an accelerated laundering machine. After washing for 120 min (equal to 4 standard tests) and drying, it could keep stable electrical conductivity for DI water that invisible visual change was found (Fig. S14). Although a slight increase in relative resistance change was observed for the soap solution, it could return after 6 h storage, indicating the good stability of the SaLM-SeCNT ink on the textile (Fig. 3h, inset). Besides, negligible change in the air permeability was obtained between the pure textile and printed e-textile varying no. of WPU–HEC coating (i.e., 0, 2, and 5 times), which are 2.097, 2.115, 2.358, and 2.656 kPa s^−1^ m^−1^, respectively (Fig. 3i). Moreover, the wearability of the printed e-textile was confirmed by studying the adhesion strength of the pattern to abrasion. An insignificant increase was found in the electrical resistance of the printed e-textile by experiencing up to 1000 continuous abrasion tests, ensuring its strong adhesive behavior, and stable resistance strength (Fig. 3j). Consequently, the desired rheological, electrical conductivity, mechanical, and stretchability of the ink printed on textile inks together with considerable biocompatibility, water resistance, washability, air permeability, and wearability ensured the potential as smart clothing.

### Strain- and Temperature-Sensing Properties of the SaLM-SeCNT e-Textile

**Fig. 4:**
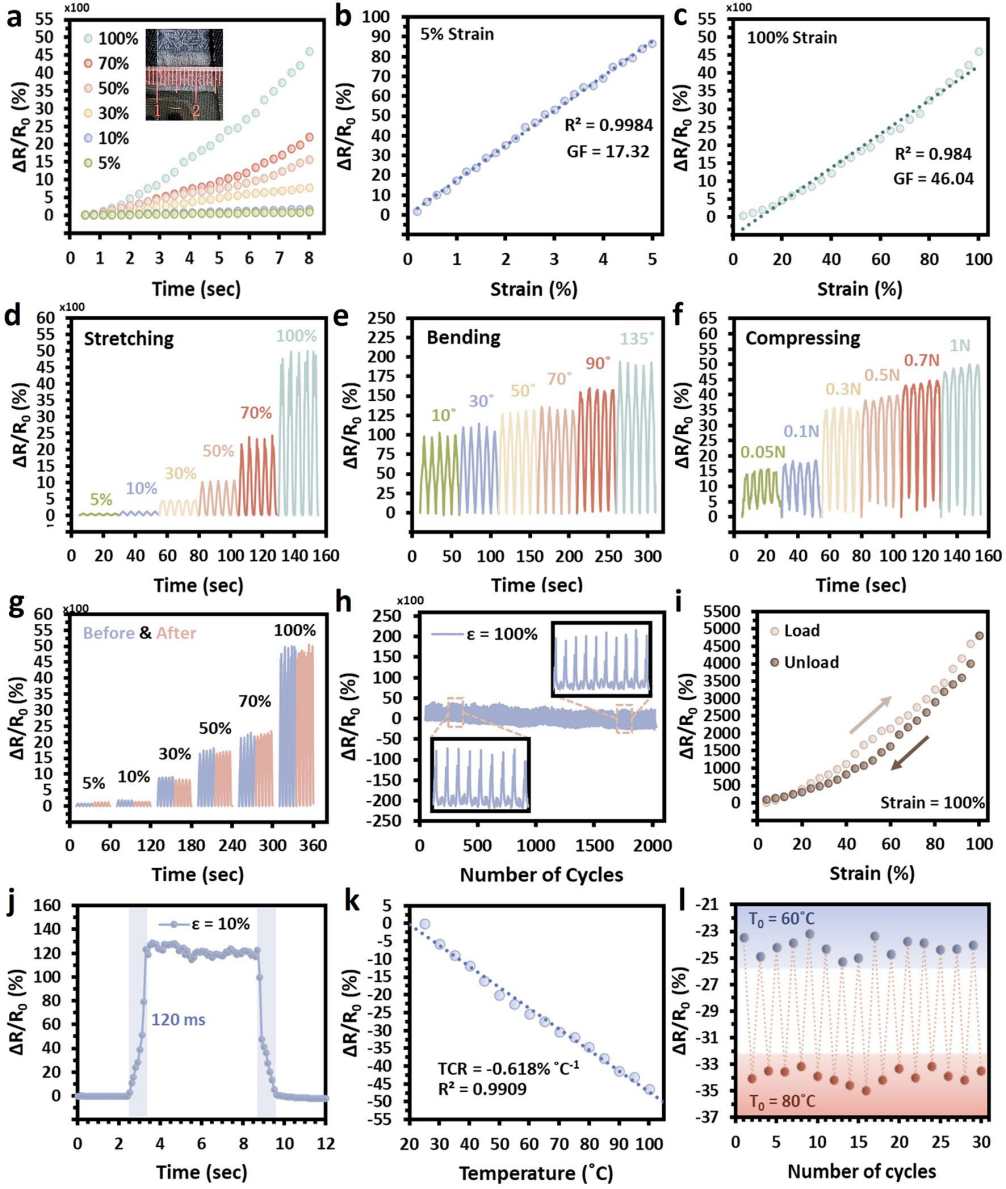
Strain- and temperature-sensing performance of the SaLM-SeCNT printed e-textile. **a** Typical relative resistance versus strain curve of SaLM-SeCNT printed e-textile under different strains. Relative resistance change and gauge factor versus applied strain within strain range of **b** 0–5% and **c** 0–100%. Time-dependent relative resistance variations of SaLM-SeCNT printed e-textile under various cyclic **d** strains, **e** bends, and **f** compressions. **g** Comparison of the stability of the relative resistance change before and after several applied mechanical strains. **h** Variation of the relative resistance change of the printed e-textile in 2000 stretching/ relaxation cycles between 0 and 100% strain. **i** Load–unloading hysteresis curve of the SaLM-SeCNT printed e-textile at the strain of 100%. **j** Response curve of the SaLM-SeCNT printed e-textile with an applied strain of 10% shows a response time of 120 ms. **k** Normalized relative resistance changes of the SaLM-SeCNT printed e-textile upon increasing temperature from 25 to 100 °C. **l** Repetitive temperature-discrimination ability of the e-textile during repeatedly cooling and heating between 60 °C and 80 °C

On the basis of the above properties, the printed e-textiles could be utilized as strain sensors, and their electromechanical performance was investigated. A screen-printed e-textile strain sensor with a 20 × 2 mm^2^ pattern was presented using the 10 wt% of SaLM-SeCNT ink (Fig. 4a, inset). The SaLM-SeCNT sensor was stabilized before the experiment by > 20 pre-stretching under 50% strain and then kept at an ambient condition for 12 h. As seen in Fig. 4a, a steady increase in the relative resistance change (Δ*R*/*R*_0_) is found, following a monotonic increment trend in resistance with a maximal strain of up to 100%. Figure 4a measures the sensing curve of the SaLM-SeCNT e-textile sensor in a small and a large region with high linearity corresponding to GF (namely gauge factors, GF = (Δ*R*/*R*_0_)/Δ*ε*, where *R*, *R*_0_, and *ε* represent the real-time resistance, the initial resistance, and the applied strain). The GF was 17.32 for the strain range within 5% with a linearity of 0.9984 (Fig. 4b) and 46.04 for 0–100% with a linearity of 0.984 (Fig. 4c), demonstrating the flexibility and sensitivity of the SaLM-SeCNT e-textile sensor in both small and large range. The linear strain-sensing properties of the SaLM-SeCNT e-textile can be explained by the stable interfacial mechanism of the conductive ink and the hierarchical fabric structure, as illustrated in Fig. S15. According to the SEM images addressed previously, extra-small and compacted cracks appeared on the conductive ink surface at a strain of 50%, followed by invisible increases in crack size under 100%. Such continuous and stable crack generation was relevant to the linear increase in GF upon stretching because of the strong adhesive and interconnect of the printed conductive pattern, resulting in precise sensitivity.

The durability and reproducibility were ensured under cyclic stretching and releasing versus applied strain (i.e., 0–100%), bending angle (i.e., 10–135°), and compression load (0.05–1 N) (Fig. 4d–f). A stable result was noticed in each cycle with a maximum relative resistance change at the highest applied change. The e-textile could also maintain a reliable sensitivity after several mechanical stretches at each strain level (Fig. 4g) and an unobvious change over 2000 stretching and relaxation cycles under a strain of 100% at a working rate of 60 mm s^−1^ (Fig. 4h). Moreover, the response of the e-textile sensor exhibits a relatively low hysteresis (Figs. 4i and S16), which is crucial for practical applications. Additionally, the SaLM-SeCNT sensor also gave a fast response to the applied strain of 120 s under a strain of 10% at a strain rate of 60 mm s^−1^ (Fig. 4j). It was indisputably conducive to monitoring real-time peoples’ fast and complex movements.

The thermosensation properties of SaLM-SeCNT e-textiles were also monitored under a heating condition. The temperature coefficient of resistance (TCR) was quantified in which formula *R*_0_ is the initial resistance of SaLM-SeCNT e-textile at 25 °C, and ∆*R* is the resistance change corresponding to temperature change ∆*T*. Figure 4k markedly shows a TCR value of − 0.618% °C^−1^ with high linearity of relative resistance change (0.9909) when the temperature is constantly changed from 30 to 100 °C. As expected, the appreciable negative TCR behavior was attributed to the variable-range hopping transport mechanism between SaLM and SeCNTs, which facilitated the mobility of charge carriers and compact pathways at higher temperatures, leading to a decrease in interface resistance (Fig. S17) [57, 58]. The interaction between SaLM and SeCNTs not only enhanced the electrostatic interaction and adsorption but also enhanced the reversible transport properties of the materials. The temperature-discrimination capacity of the e-textile is depicted in Fig. 4l. The relative changes in electrical resistance were manifested as visible divergence under alternating 60 °C and 80 °C. Obviously, the output signal showed high repeatability and stability of the SaLM-SeCNT e-textile. Therefore, the practical feasibility of the temperature response made the SaLM-SeCNT e-textile possible for wearable sensing devices.

### Electric Output Performances of the SaLM-SeCNT e-Textile TENG

**Fig. 5:**
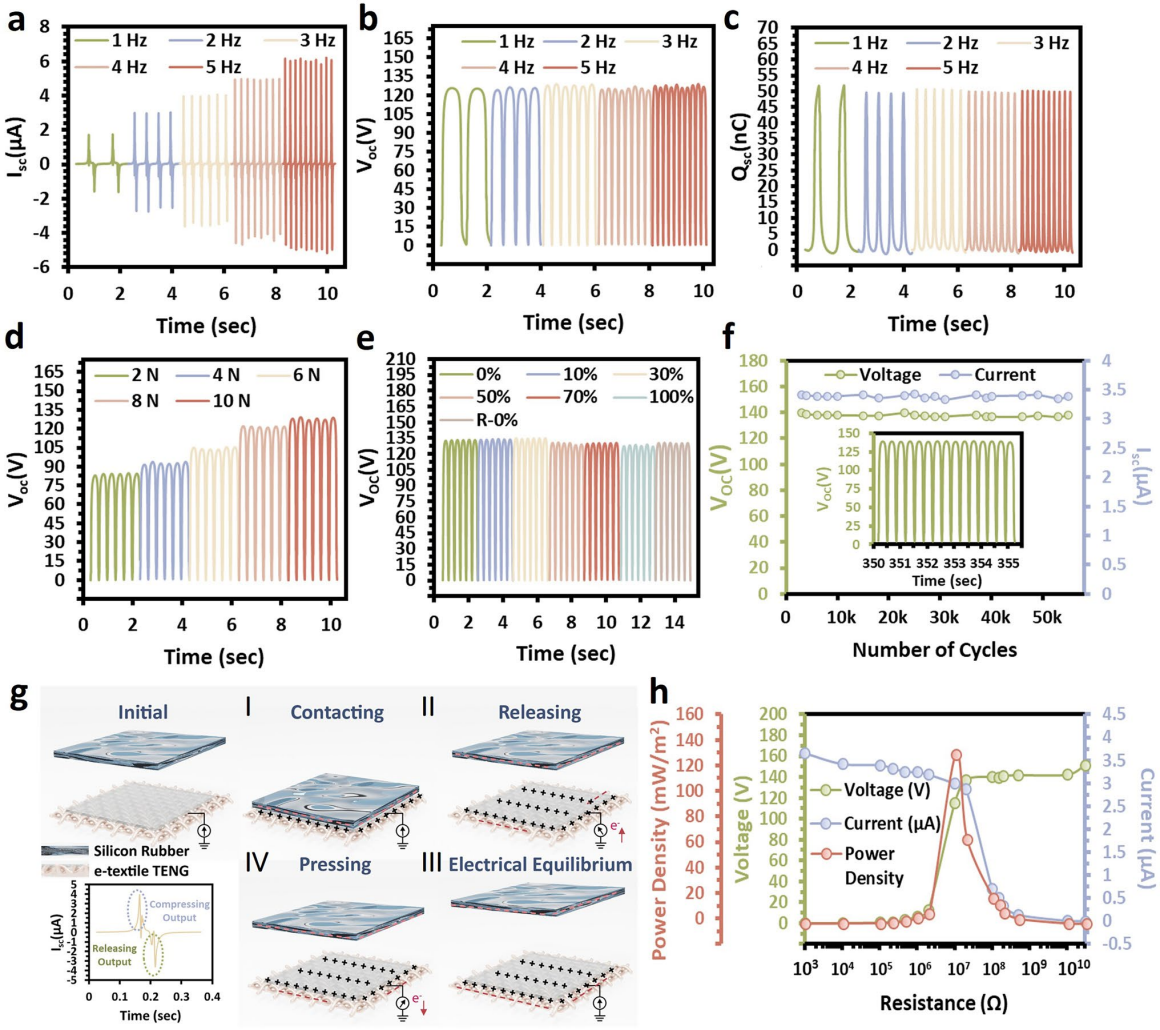
Working mechanism and electrical output performance of the SaLM-SeCNT e-textile TENG. **a** *I*_SC_, **b** *V*_OC_, and **c** *Q*_SC_ of the SaLM-SeCNT e-textile TENG under different frequencies (1–5 Hz). **d** *V*_OC_, of the SaLM-SeCNT e-textile TENG under different loading. **e** *V*_OC_, of the SaLM-SeCNT e-textile TENG at various tensile strain levels (0–100%) and recovery states. The initial length is 20 mm, and the contact frequency is 3 Hz. **f** Durability and stability test of the SaLM-SeCNT e-textile TENG under continuous impact for 50,000 cycles. The insets exhibit detailed *V*_OC_ cycles. **g** Schematic diagrams and working mechanism of the SaLM-SeCNT e-textile TENG. **h** Dependence of the output voltage, output current, and output power density with the various external load resistance

In addition to the excellent flexibility of the thin WPU–HEC layer as a protective coating [59], it also exhibits a remarkable ability to gain or lose electrons when in contact with different materials, allowing it to be the triboelectric layer as a single-electrode textile TENG [60]. In this work, the e-textile TENG (25 × 25 mm^2^) was prepared by introducing a copper wire between the screen-printed SaLM-SeCNT conductive pattern and the WPU–HEC coating. The electrical performance under various frequencies (1, 2, 3, 4, and 5 Hz) of contact separation is also examined in Fig. 5a–c. *I*_SC_ was increased from 1.9 to 6.2 μA, while there was no significant difference in both the *V*_OC_ and *Q*_SC_ (≈ 130 V and 50 nC). This phenomenon can be explained theoretically by Gauss theorem [61] which defines no response of voltage and static charge density to the frequency. Subsequently, the short-circuit current of the e-textile TENG is determined based on Maxwell’s displacement current [62] that the charge density shows a proportional result to the output current on the static charge density and the speed at which the two dielectrics contact/separate, leading to a constant result. The electrical output values exhibit an increasing trend under various impact forces (2, 4, 6, 8, and 10 N) in Fig. 5d in which the maximum output (~ 130 V) is obtained at the force of 10 N owing to the effective contact electrification effect. Similar results were also found in its *I*_SC_ and *Q*_SC_ (Fig. S18). The changes in electrical signal demonstrated that the electrical fabric was highly sensitive to the strength and frequency of external impact forces, so it could also serve as a sensor to monitor the changes in external forces while collecting energy.

Considering the practicability of the e-textile TENG, the electrical performance and work stability were investigated. The stable and durable electrical output is an important requirement for wearable triboelectric fabric, and thus, the electrical output at various tensile conditions was systematically investigated under the force of 3 Hz/10 N. There was almost no reduction in *V*_OC_ (Fig. 5e) and a slight reduction in I_SC_ output at different strain levels (Fig. S19). This could be attributed to the decrease in connection efficiency of the electrode and charge induction efficiency when the electrodes became sparse in the unit area during stretching. And when the fabric was stretched by 100%, the resistance of the electrodes might increase certainly which might also be the reason for the decreased output. More importantly, the output performance of e-textile fabrics could return to its original state after repeated stretch-release from 0 to 100% (Fig. 5e). Besides, the *V*_OC_ and *I*_SC_ of the device under 50,000 repeated working cycles at 5 Hz were also tested (Fig. 5f). It is observed that the signal value of the device was similar to the initial value without visible reduction.

Figure 5g systematically illustrates the structural design and working principle of the e-textile TENG in which the silicone rubber and WPU–HEC layer are determined as the negative and positive triboelectric materials, respectively, owing to their difference in relative polarity. When the two subjects contact, silicon rubber carries negative charges because of the strong negative charge-capturing ability, while a similar amount of positive charge is generated on the surface of the WPU–HEC layer to keep the potential in balance (Stage I). Upon release, the unscreened positive charges will induce the accumulation of negative charges in the SaLM-SeCNT conductive interface. Meanwhile, the instantaneous electrons will flow from the ground to the printed conductive layer (Stage II). When the silicon rubber and the WPU–HEC layer are fully separated, the potential in the textile TENG system reaches an equilibrium and the flow of free electrons will also terminate (Stage III). During a new surface approach, the electrons will return from the printed conductive layer to the ground, indicating a reversed operation mechanism (Stage IV). By repeating the contact-separation procedure mentioned above, an alternating electrical signal can be generated.

To further study the electric output performance of the e-textile TENG, the voltage, current, and output power density of the optimized device were discussed under a series of external resistances. As the resistance was increased from 1 KΩ to 10 GΩ, the voltage of the fabrics exhibited an obvious increase to a high value, while the current displayed an opposite trend. The output power was calculated according to the formula *P* = *I*^*2*^*R* (where “*R*” represents the load resistance and “*I*” represents the output peak current through the external load), and the instantaneous power density showed a maximum value of 162 mW m^−2^ at a load resistance of 1.5 × 10^7^ Ω (Fig. 5h). Table S2 shows that the power density of the SaLM-SeCNT e-textile TENG was larger than the reported ink-based flexible TENGs using different ink printing techniques.

### Practical Application of SaLM-SeCNT Ink in Wearable Sensing Devices

**Fig. 6:**
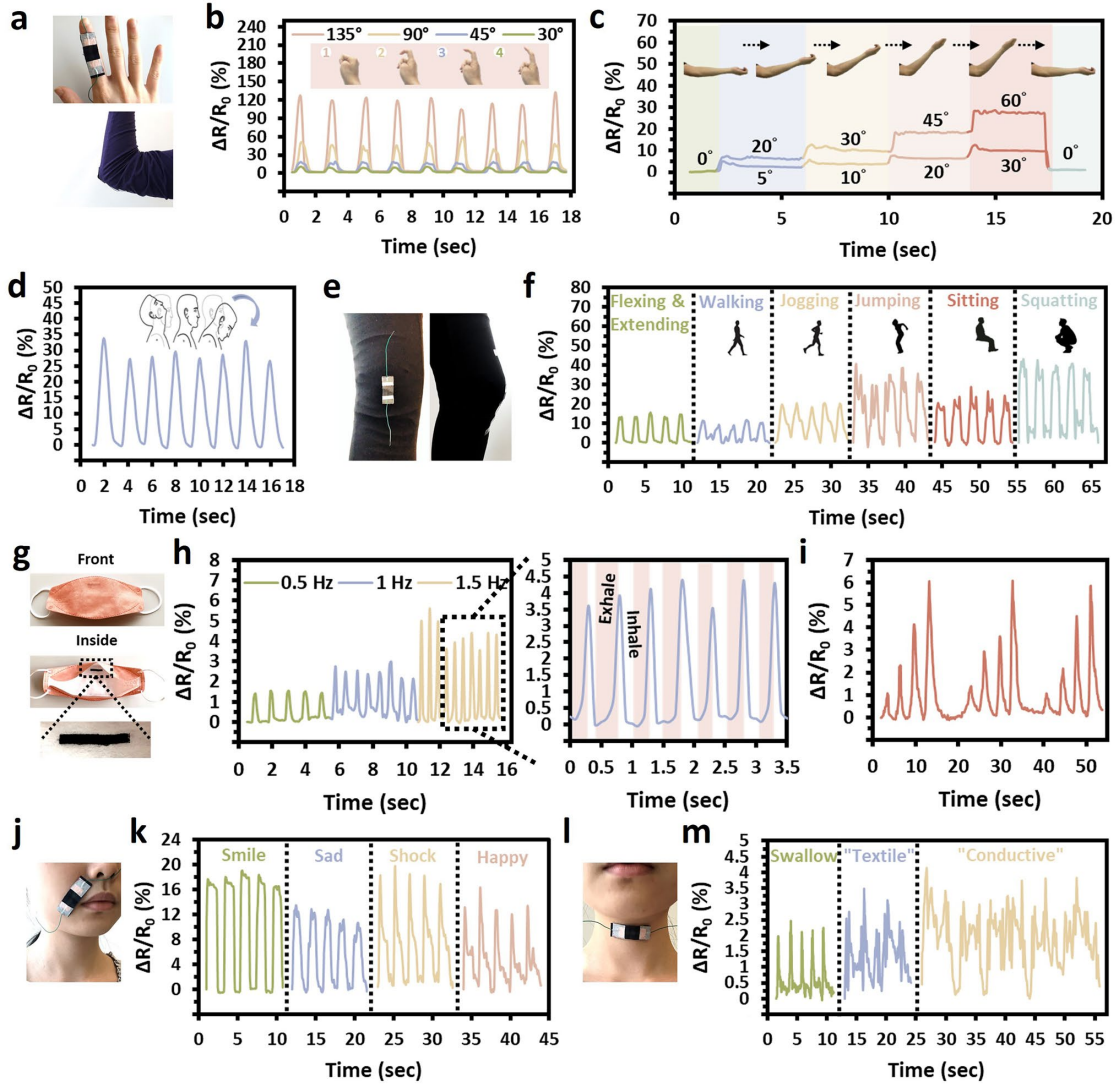
Sensing applications of wearable SaLM-SeCNT printed e-textile for detecting various human motions, breathing, and speaking. **a** Photographs of hand and elbow attached with SaLM-SeCNT printed e-textile. Relative resistance changes of **b** finger, **c** elbow, and **d** neck bending at different angles. **e** Optical image of sensor assembles on commercial jeans for knee joint sensing. **f** Corresponding signals of flexing/extending, walking, jogging, jumping, sitting, and squatting. **g** Photographs of a commercial protective mask with SaLM-SeCNT ink printed inside and **h** relative resistance changes of the sensor monitoring breath at different frequencies. **i** Corresponding signal of continuous respiration modes from light to deep breathing by attaching to the abdomen. **j** Photograph of the SaLM-SeCNT printed e-textile attached near the mouth and **k** responsive of sensor signals in monitoring tiny muscle movements of a smile, sad, shock, and happy. **l** Photograph of the SaLM-SeCNT printed e-textile attached at the throat and **m** corresponding signals of swallowing and phonation when the wearer spoke: “textile” and “conductive”

By virtue of its sensitivity and wearability, the SaLM-SeCNT printed e-textile showed potential applications in serving as wearable sensors for full-range recognition of human activities. Testifying the potential applicability of biomonitoring, the e-textile sensor was fabricated by direct printing the SaLM-SeCNT ink onto different commercial garments (such as gloves, T-shirts, jeans, and compression bends) and on different parts of the body for real-time motion monitoring and multiple physiological signal detection. As depicted in Fig. 6a–f, efficient and precise sensitivity can be seen to detect changes in relative resistance caused by the precise tracking of the different joint bendings. A steady increase or decrease in relative resistance was observed under continuous finger motion (Fig. 6b). High stability in relative resistance was also found in which a similar value was maintained by continuous bend holding at the same angle, which displayed the instantaneous and accurate measurement of joint movements. The SaLM-SeCNT printed e-textile could also be applied for distinguishing and monitoring the joint supple through larger deformation of the wrist (Fig. S20), elbow (Fig. 6c), and neck (Fig. 6d) at different angles. The relative resistance was correspondingly increased with a continuous increase in joint angle, presenting a unique step-like feature. It was notable that when the elbow returned to a flat state, the signal was fully restored (Fig. 6c). With its high wearability and sensitivity, such an e-textile sensor came in handy for repairing and treating joint injuries. Furthermore, SaLM-SeCNT ink-printed commercial conductive jean/tight (Fig. 6e) was developed to detect and discriminate different motion conditions of the knee, such as extending/flexing, sitting, squatting, jumping, walking, and jogging (Fig. 6f). It could be observed that the magnitude was increased due to the large deformation caused by the movement.

Additionally, rapid and precise small-scale signal sensing was also demonstrated such as breathing, facial expressions, speaking, and pulse. SaLM-SeCNT ink was printed on the commercial mask gently (Fig. 6g) and elegantly to detect real-time respiration rate and depth. Figure 6h shows the exhale and inhale of respiration cycles and repeatable sensing cycles are also found by increasing the frequency and deepness (Fig. 6i). The results manifested the breathing conditions under relaxation and exercise. Accurate sensing could also be addressed in facial expressions and speaking (Fig. 6j–m). The printed e-textile was attached to the skin near the mouth and cheek (Fig. 6j) to collect weak muscle change induced by different facial expressions. For example, the relative resistance changes versus the time of the facial muscle stretching and relaxing could be accurately recorded from a poker face to a smile, a sad face, a shocked face, and cheek laughing (Fig. 6k), respectively. A similar way is utilized to recognize tiny epidermis and throat muscle changes during swallowing and speaking various words “textile,” and “conductive” by locating the e-textile on the neck position, as shown in Fig. 6l-m. The above signal patterns with good repeatability revealed that the SaLM-SeCNT printed e-textile has satisfied applications in the field of phonation recognition.

Furthermore, the SaLM-SeCNT e-textile could be applied to measure body and environment temperature. To quantitatively reveal human skin temperature, the SaLM-SeCNT ink-printed e-textile was installed from a room condition to a healthy adult underneath. As shown in Fig. S21, when the device is attached securely to the human underneath from the environment temperature, a shape relative resistance change of about − 7.59% is shown. It distinguished the temperature change and difference between the room condition (25 °C) and body temperature at the static stage (36.4 °C). Then, the relative resistance change was continuously decreased to − 8.78% if the subject started to exercise which meant that the temperature of the subject’s body rose to 37.8 °C. Such sensing response of narrow physiological temperature ranges potentially offered the e-textiles in tracking workout or fever conditions. Beyond that, the surrounding temperature could be monitored by storing in ice (− 30 °C) and heat (50 °C) environment (Fig. S22). This temperature monitoring should be used as thermometers to actively measure the body temperature of patients with fever and workout monitoring, as well as fire alarm systems for safety applications.

### Practical Application in Energy Harvesting and Self-powered Pressure Sensing

**Fig. 7:**
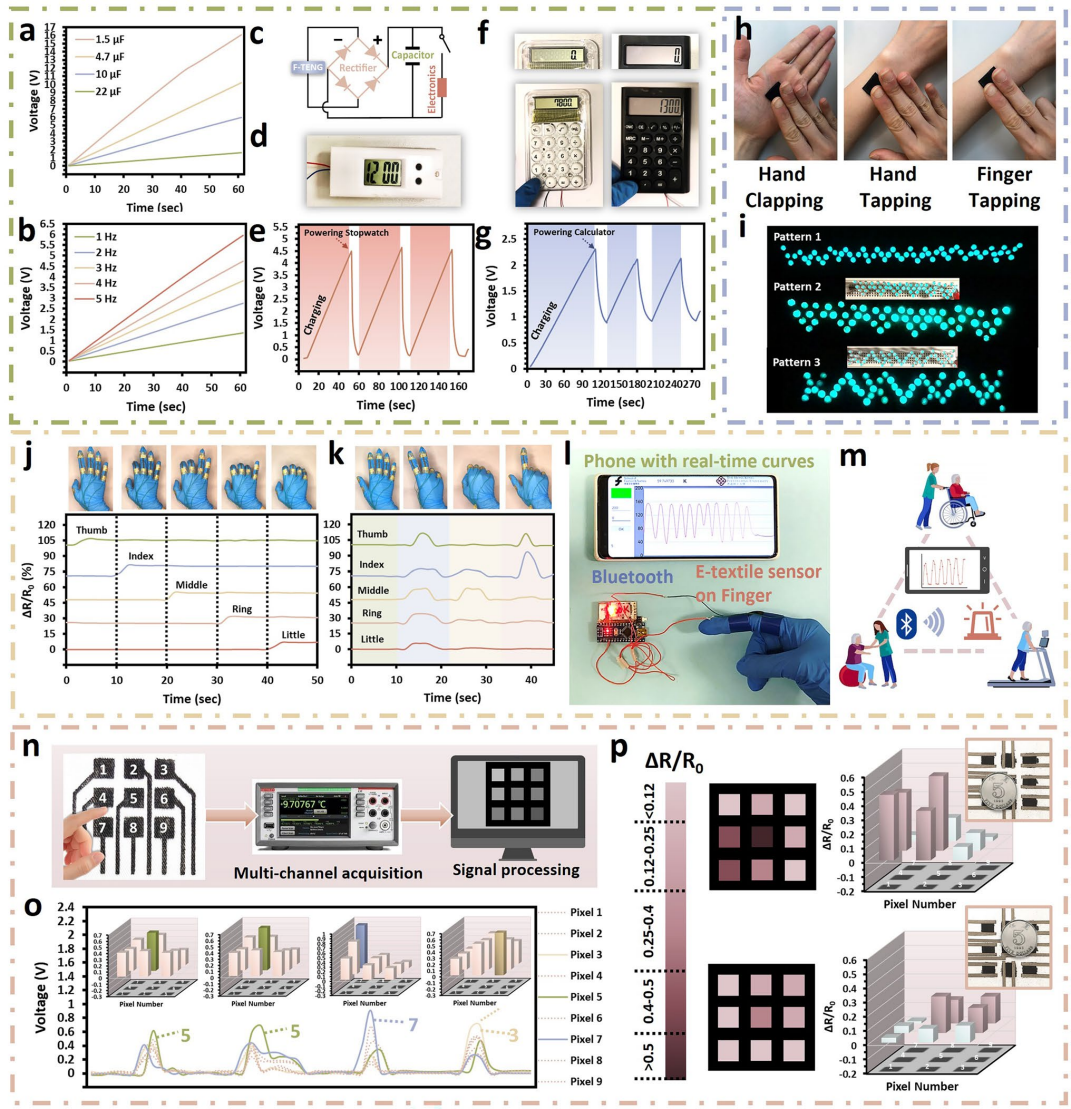
Practical applications of the SaLM-SeCNT printed e-textile in energy harvesting, power sourcing, and self-powered sensing. Charging ability of the SaLM-SeCNT printed e-textile TENG **a** for different capacitor capacities and **b** at different frequencies from 1 to 5 Hz as a power source supply. **c** Working circuit of the self-powered system. Demonstration of self-powered electronic devices: **d, e** digital stopwatch with voltage curves of 10 μF storage capacitors and **f**, **g** digital calculator with voltage curves of 22 μF storage capacitors from hand tapping. **h**, **i** Photograph of the LED lighting from hand clapping, hand tapping, and one finger tapping. **j** Photographs and Δ*R*/*R*_0_ of SaLM-SeCNT printed smart glove with different gestures. **l** Demonstration of the external connections of the e-textile sensors with wireless detection. **m** Potentials of the sensors used in health monitoring, rehabilitation, and warming applications. **n** Schematic diagram of the pressure-sensing system, which includes e-textile patterned with 3 × 3 pixels, multichannel acquisition, and signal processing. **o** Reliable sensing signals of the patterned e-textile TENG in tapping different pixels in the order of 5, 5, 7, and 3. **p** Pressure distribution and the relative resistance change of the pattern e-textile sensor in sensing the object in different locations

Multifunctional properties of the e-textile were demonstrated by powering small electronic devices with the aid of DC-powered circuits and LEDs as an energy harvester. A patterned triboelectric textile TENG was fabricated by printing the SaLM-SeCNT ink. Capacitors with diverse capacitances of 1.5, 4.7, 10, and 22 μF can be rapidly charged to 16, 10.2, 1.7, and 1 V in 60 s, respectively, as presented in Fig. 7a, while different charging rates for a 10 μF capacitor at multiple charging frequencies are also exhibited in Fig. 7b. A faster-charging speed can be found in the capacitors with a lower capacitance and higher working frequency at the same potential. The e-textile TENG was capable of storing energy in capacitors to drive wearable/portable electronics and the working circuit was addressed (Fig. 7c). When continued contact-separation was applied to the e-textile TENG, the capacitor voltage was increased to 4.5 V in 50 s (10 μF), and 2.2 V in 100 s (22 μF), respectively, which was enough to drive the electronic stopwatch (Fig. 7d, e) and calculator (Fig. 7f, g) with a repeatable charged-discharged record more than 3 times. Concerning real-time energy applications, the e-textile TENG was employed in powering an electronic calculator for simple calculation (Video S1). This fact indicated the proposed e-textile TENG has the potential in serving as a self-powered energy source for different low-power wearable/portable electronic devices. Besides, the SaLM-SeCNT e-textile TENG could easily power LEDs to full brightness in different patterns by harvesting the mechanical energy of hand tapping (Fig. 7h, i). Video S2 also demonstrates its ability as an energy supply in powering more than 100 LEDs by hand tapping. These above demonstrations sufficiently proved the ability and feasibility of the SaLM-SeCNT e-textile to drive microelectronics without any external power source by converting the mechanical energy of human motion into electrical energy.

As a consequence of remarkable sensing results, it has been supplemented in real-time detections. For example, a SaLM-SeCNT printed e-textile glove could be developed and each finger was connected with an individual channel. Repeatable and significant signals were collected under different hand motions such as one-by-one finger bending and holding (Fig. 7j), and hand postures (Fig. 7k), simultaneously. The sensing system composed of the SaLM-SeCNT printed e-textile, a digital multimeter with a Bluetooth module, and a mobile phone was established to demonstrate the feasibility of health monitoring. Generally, human activities were collected and processed through wearable flexible textile sensors connected with a digital multimeter, and an app on the mobile phone could monitor the resistance change in real-time. As shown in Fig. 7l and Video S3, Supporting Information, a systemic increase can be seen in the index finger and elbow-bending tests in which the resistance signals operated steadily with the increase of joint angle. If the bending angle increased and achieved a certain level of the threshold value, an alarm or warning allowance could be set through the mobile app. Therefore, a potential application might be used in monitoring workout performance for athletes, in the rehabilitation exercise, and the intensive care unit to continuously monitor patients with limited mobility or safety warnings for patients (Fig. 7m).

In addition to strain-sensing, the promising electric output and self-powered characteristics of the SaLM-SeCNT printed e-textile could offer its capabilities of recognizing the tactile trajectory and detecting the pressure distribution. The flow chart of the intelligent control system is illustrated in Fig. 7n in which a fabric with 3 × 3 pixels printed conductive pattern numbering from 1 to 9 is connected mutually to independent channels. When the printed pixel was touched or pressed, respectively, it generated relative output voltage signals and the corresponding channels will capture the trajectory of the touching actions in the term of electronic signals by the multichannel data acquisition method. The real-time voltage signals of the 9 pixels could be distributed simultaneously in response to the mechanical forces which is a desirable feature in real-time detection. As displayed in Fig. 7o, significant voltage signals can be seen by touching a specific tactile sensor array (i.e., 5, 5, 7, 3) in which the touched pixel gives the highest relative voltage output whereas lower signals are found in the surrounding pixels. The insets addressed the pressure distribution of the 9 pixels under continuous finger touching. Besides, the patterned e-textile could achieve the mapping of external forces based on relative resistance change. The precise detection of each pixel is ensured in Fig. S23 that a significant signal of the touched pixels could be seen clearly. Thus, the patterned e-textile could distinguish the placement of a 5-dollar coin in different locations with corresponding relative resistance change (Fig. 7p). Figure S24 also confirms its pressure-sensing ability in differentiating different objects (e.g., sponges, pens, and coins) with replicable pressure signals. According to the demonstration, different circuit patterns could be simply printed on commercial garments for multiple sensing (e.g., health monitoring, pressure tracking, multitouch devices, and temperature sensing) and powering applications that performed high feasibility, breathability, comfortability, biocompatibility, and ease of fabrication.

## Conclusion

In summary, by taking advantage of biopolymers and gelatinous protein, SeCNT-wrapped SaLM nanoparticles were naturally obtained with strong interactions and crosslinkings which could be uniformly dispersed in water and served as a stable conductive ink for printable wearable electronics. Owing to the remarkable stabilization effect, it showed high adaptability and was compatible with various flexible substrates of paper, polymeric films, and textiles by ink-writing and printing technologies. The fabricated e-textile not only achieved integrated characteristics, including significant mechanical and electric performance, linear sensitivity, colloidal stability, wearability, wearing comfort, durability, and biocompatibility but also promoted additional aesthetic features and ease of production. As proofs of concept, multifunctional applications were demonstrated including motion and breath monitoring, pressure tracking, self-powered sensing, and energy harvesting. In this regard, it can foresee that this green-based SaLM-SeCNT aqueous ink could bring a new concept to the development of eco-friendly conductive ink and wider the application of biocompatible flexible electronics.

## Supplementary Information

Below is the link to the electronic supplementary material.

Supplementary file1 (PDF 1457 KB)

Supplementary file2 (MP4 13867 KB)

Supplementary file3 (MP4 15179 KB)

Supplementary file4 (MP4 16509 KB)
