# Supplementary material for: Naturally Crosslinked Biocompatible Carbonaceous Liquid Metal Aqueous Ink Printing Wearable Electronics for Multi-Sensing and Energy Harvesting
Source: Nanomicro Lett. 2024 Mar 11;16:149. doi: 10.1007/s40820-024-01362-z (PMC10928061; doi:10.1007/s40820-024-01362-z)
Supplement: Supplementary file 1 — Supplementary file1 (PDF 1457 KB) [file 40820_2024_1362_MOESM1_ESM.pdf]

Supporting Information for

Naturally-Crosslinked Biocompatible Carbonaceous Liquid Metal Aqueous Ink Printing Wearable Electronics for Multi-Sensing and Energy Harvesting

King Yan Chung[1], Bingang Xu[1,]*, Di Tan[1], Qingjun Yang[1], Zihua Li[1], Hong Fu[2]

[1]Nanotechnology Center, Research Institute for Intelligent Wearable Systems, The Hong Kong Polytechnic University, 999077, Hong Kong, P. R. China

[2]Department of Mathematics and Information Technology, The Education University of Hong Kong, Hong Kong, P. R. China

*Corresponding author. E-mail: tcxubg@polyu.edu.hk (B. Xu)

Supplementary Figures

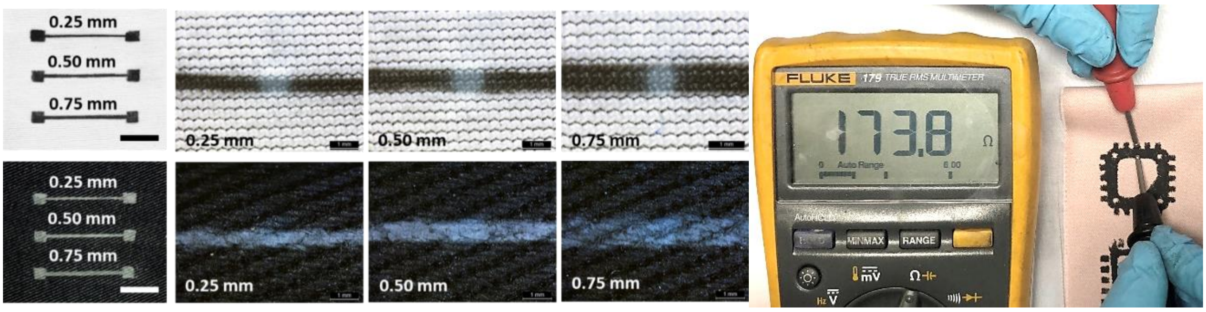

Fig. S1 Left: Optical images of the screen-printed SaLM-SeCNTs ink on knitted (Top) and woven (bottom) fabric in different diameters. Right: Optical image showing SaLM-SeCNT ink patterned fabric with considerable conductivity

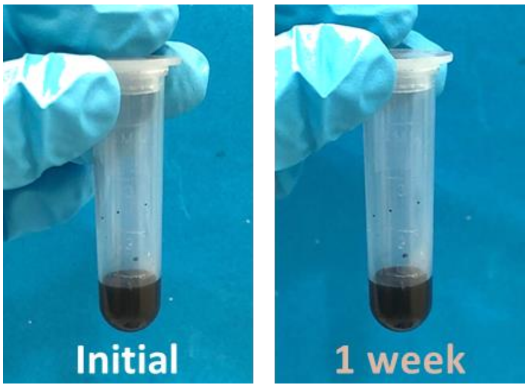

Fig. S2 Optical image showing the uniformity of the SaLM-SeCNT conductive ink in the initial states and stored for 1 week, respectively

Table S1 Comparison of different samples

Samples	wt% of SA	wt% of Se	weight ratio of LM:CNT
**LM-CNT**	/	/	LM:CNT = 1
**Ink-0.5**	0.3	0.5	SaLM:SeCNT = 0.5
**Ink-1**	0.3	0.5	SaLM:SeCNT = 1
**Ink-2**	0.3	0.5	SaLM:SeCNT = 2
**Ink-3**	0.3	0.5	SaLM:SeCNT = 3

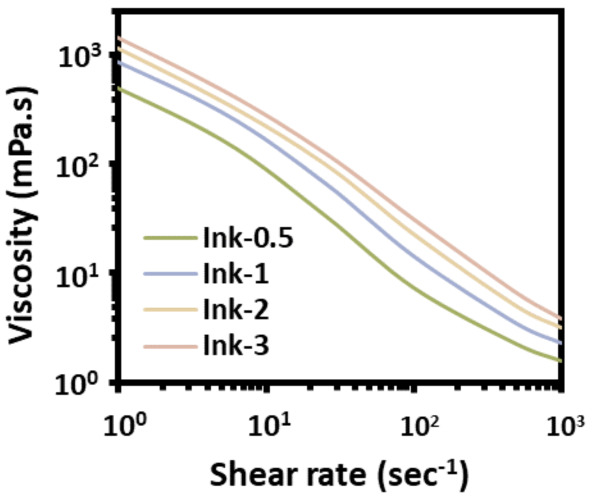

Fig. S3 Variation of the viscosity of the SaLM-SeCNTs ink with different filling ratios

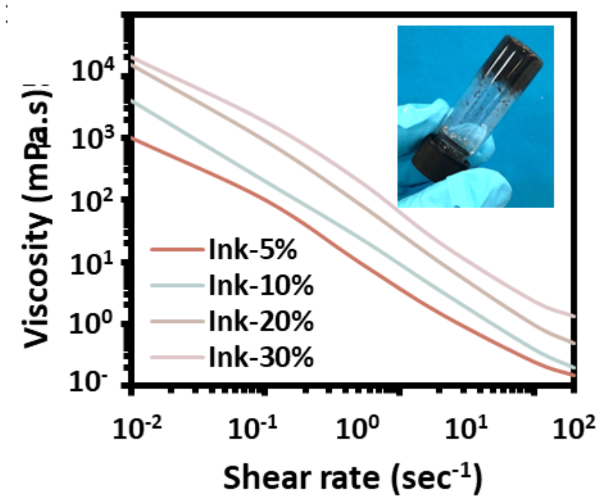

Fig. S4 Variation of the viscosity shear stress with different wt% in the ink

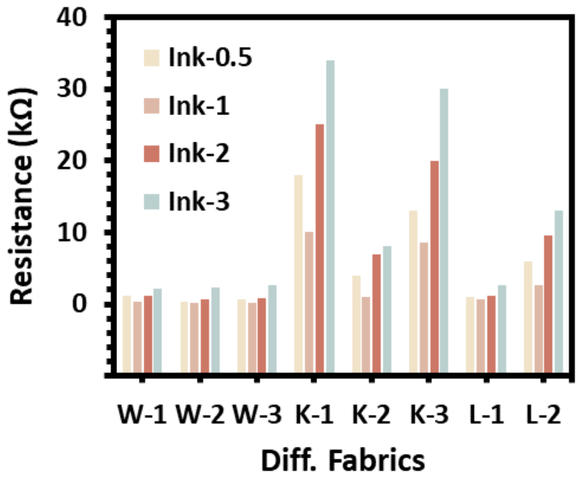

Fig. S5 The resistance of the SaLM-SeCNT ink printed on different textile materials varying from woven (i.e., W-1, W-2, W-3), knit (i.e., K-1, K-2, K-3) and lycra (i.e., L-1, L-2) fabrics

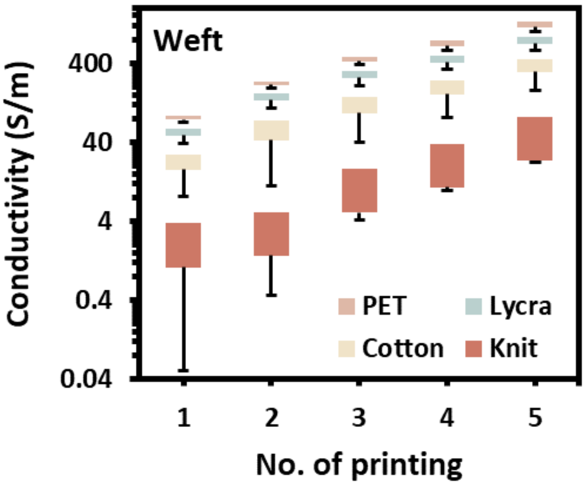

Fig. S6 The effect of No. of printing time and printing directions (weft) on the conductivity of the SaLM-SeCNT ink-printed textiles

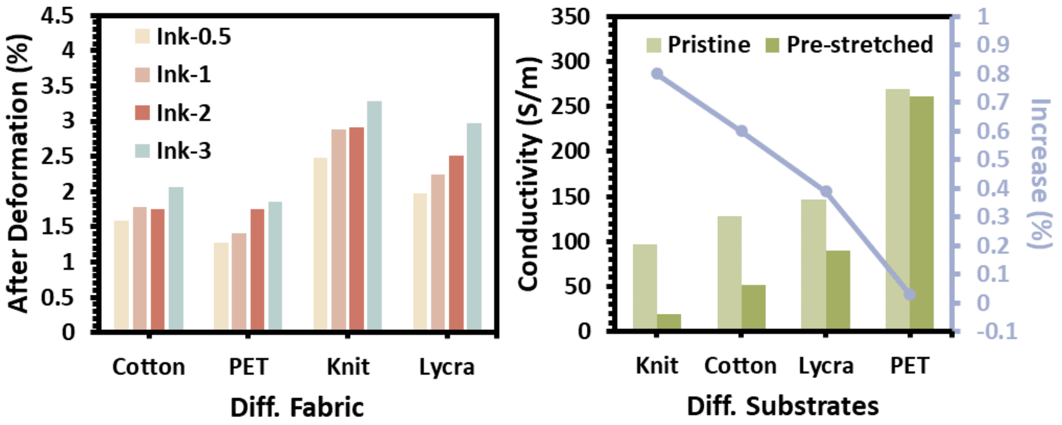

Fig. S7 The change of conductivity and increase (%) of resistance of SaLM-SeCNT ink printed on different textile materials varying from woven, knit and lycra fabrics before and after deformation

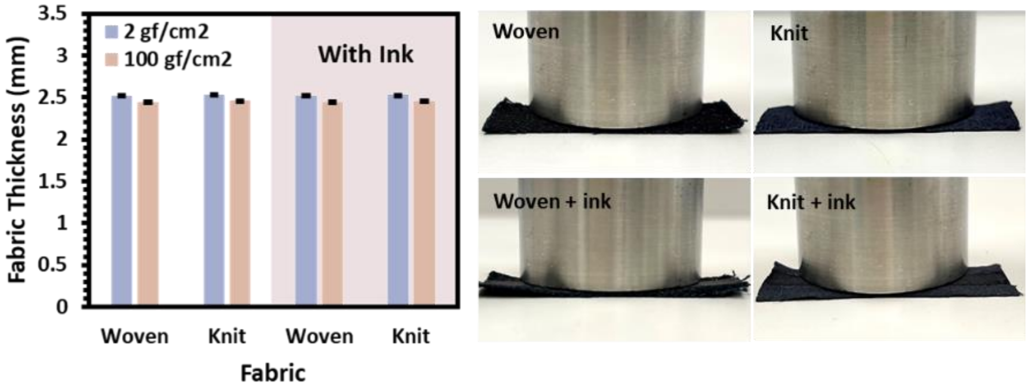

Fig. S8 The change of fabric thickness under compression and their optical images

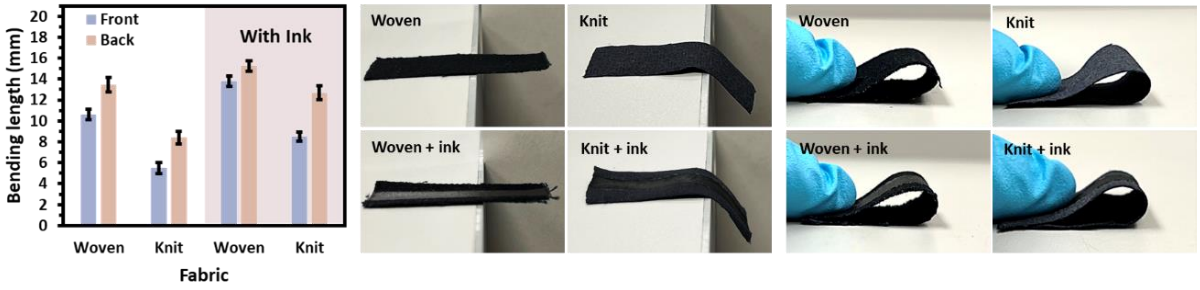

Fig. S9 The bendability of fabric and their optical images

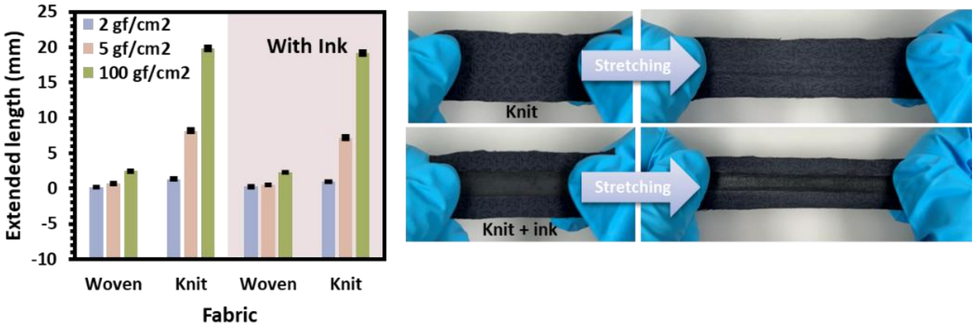

Fig. S10 Extensibility of fabric under different loadings and their optical images

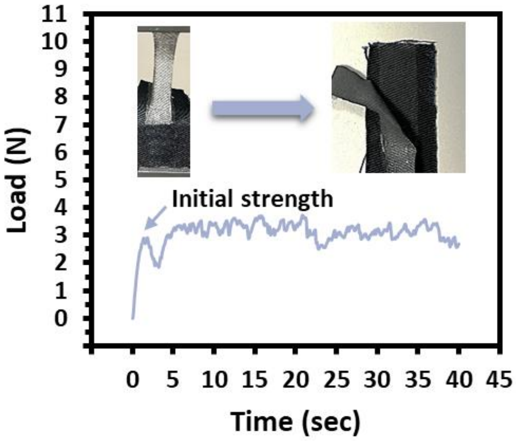

Fig. S11 The bonding strength between the SaLM-SeCNTs ink layer and textile surface

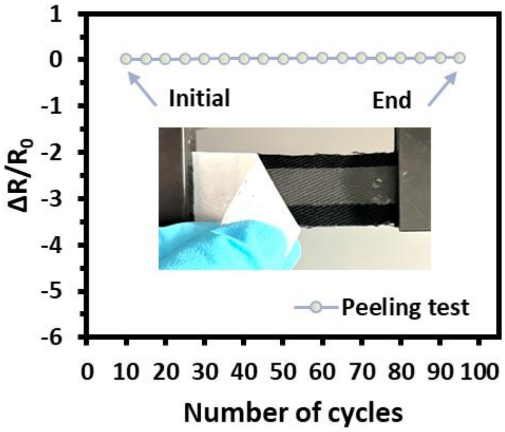

Fig. S12 Relative resistance change of SaLM-SeCNTs printed e-textile before and after 100 peeling cycles. The inset shows a digital photograph of the peeling process

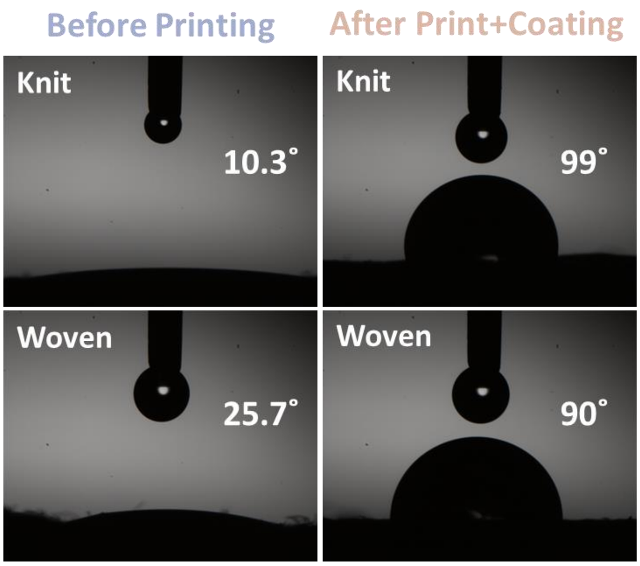

Fig. S13. The water contact angle before and after conductive ink printing and coating varying from knit and woven fabric

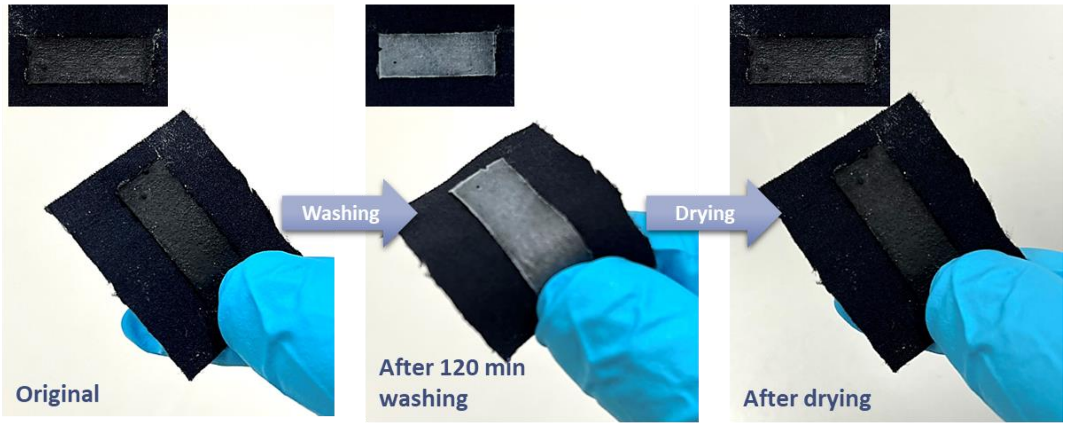

Fig. S14 Optical image of the printed e-textile before and after 120 min washing, and proper drying

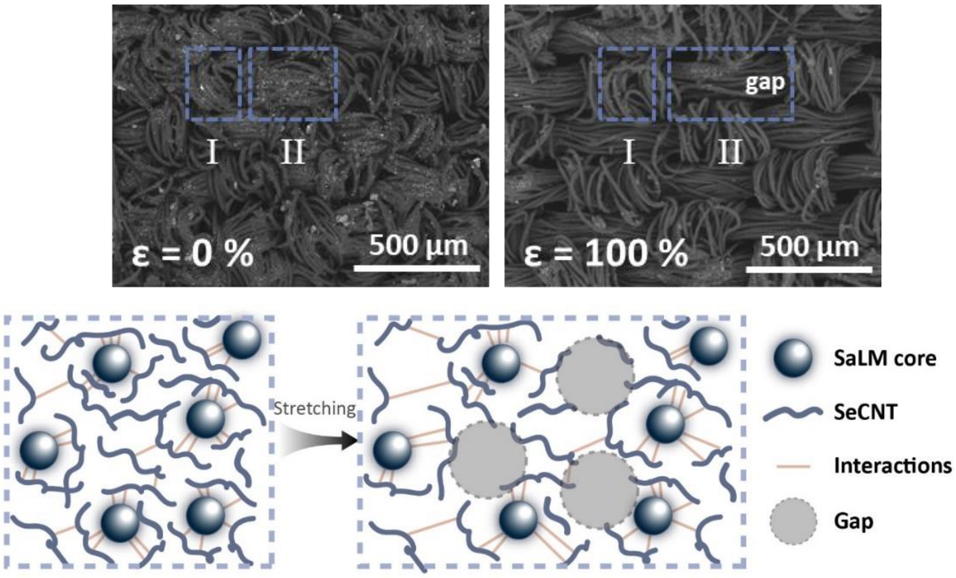

Fig. S15 Strain-sensing mechanism of the SaLM-SeCNT printed e-textile

The strain-sensing property of the SaLM-SeCNTs printed e-textiles was endowed by (1) the hierarchical fabric structure and (2) the interfacial bonding between SaLM and SeCNTs. The textile fabric as the substrate enables the stretchability and stable electromechanical properties of the sensor. Notably, the textile is constructed by continuous and dense yarns which makes it have heterogeneous properties in two distinct regions: weaving yarns (region I) and weaving gaps or extension area between the yarns (region II), as marked in Fig. S10. During the stretching process, the fabric sheet is elongated in which the weave gaps and yarn in x-direction are elongated but no large direct strain on the yarns in region II, resulting in uniform deformation. After the disposition of the conductive ink on the yarn, the good adhesiveness between them offered direct change to the resistance, as regarded as the strain-sensing performance. During stretching, the improved cross-linking and physical bonding in the water-based ink promoted the orientation of chain segments and resulted in strain-induced self-reinforcement, resulting in continuous and stable increase in resistance change.

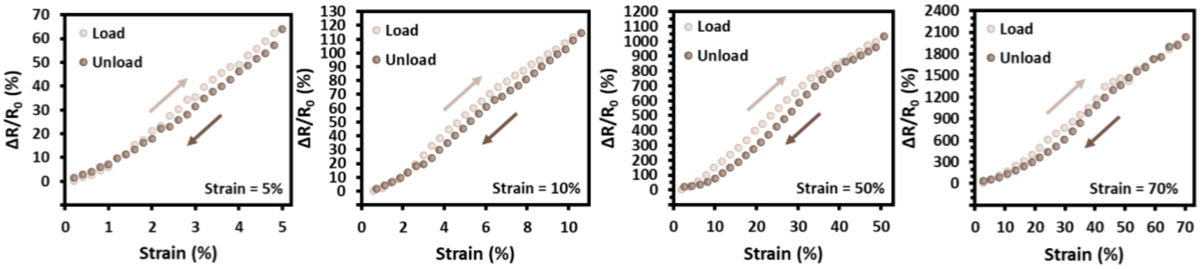

Fig. S16 Load-unloading hysteresis curve of the SaLM-SeCNT printed e-textile at the strain of 5%, 10%, 50% and 70 %

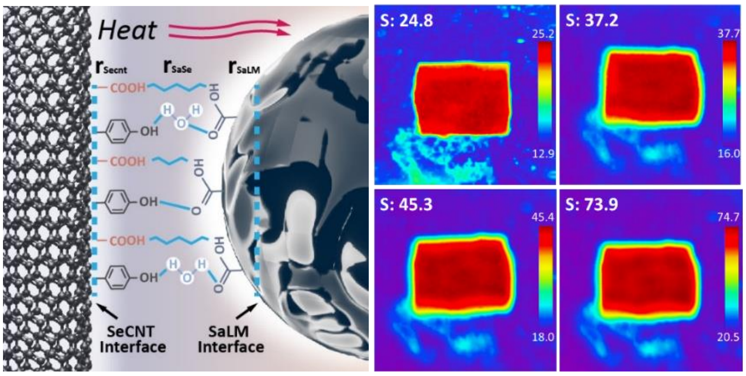

Fig. S17 Temperature-sensing mechanism of the SaLM-SeCNT printed e-textile (left) and the thermal infrared images showing the surface temperature evolution of the SaLM-SeCNTs e-textile (right)

With the high breathability and porous density of textile substrate, heat can simply transfer from one face to another face, and thus, cause a direct temperature change to the textile with SaLM-SeCNTs coating. When the e-textile is heated, the increasing temperature induces the contraction of the textile, leading to the compacted transformation of SaLM-SeCNTs conductive pathways and resistance change. The Ga_2O_3 generated by direct oxidation in the air has an amorphous structure, but has stabilized by the addition of Sa, implying the ordered organization of atoms within such a layer with photo-thermal effect. Such effect offers SaLM to absorb the applied heat, followed by transmitting or reflecting to the surrounding SeCNTs with rich functional groups. The high thermal conductivity of the SeCNTs can therefore be activated and warm up the neighboring people, generating a well-connected thermal conductive web. Subsequently, thermal conductivity mechanism mainly focuses on the impact of

interfacial bonding effect, in which the interfacial thermal resistance (r_{int}) between SaLM and SeCNT inside the system consists of three components:

$$r_{\text{int}} = r_{\text{Secnt}} + r_{\text{SaSe}} + r_{\text{SaLM}} \qquad \text{(S1)}$$

Of these sources of interfacial resistance, the value of r_{SaSe} should be smaller because of the strong bonding between SaLM and SeCNT. The long and well-aligned SeCNTs are usually of high thermal conductance and contribute negligibly to the whole thermal interface resistance which refers to r_{Secnt}. Consequently, the r_{SaLM} plays an important role which can originate LM droplets in uniform size with negatively charged, leading to a highly ordered connection with SeCNTs with positively charged. Therefore, the functional groups on both fillers exhibit rather strong electrostatic interaction which constructs effective molecular thermal linkers to decrease the r_{int}. Consequently, at initial temperature status, SeLM and SaCNT are stably distributed in the composite with acceptable spacings. Upon increasing temperature, the SaLM and SeCNTs are stretched to full expansion and decrease the network distance in between, attributing to the increasing of conductive pathways and the reduced resistance value. As a result, the thermal conductivity of the SaLM-SeCNTs e-textile could be attracted to two aspects: (1) the high intrinsic thermal conductivity of the SaLMs and SeCNTs; and (2) the formation of a well-interconnected thermal conductivity network in the whole composite region due to the uniform dispersion, resulting in stronger interfacial and electrostatic reaction.

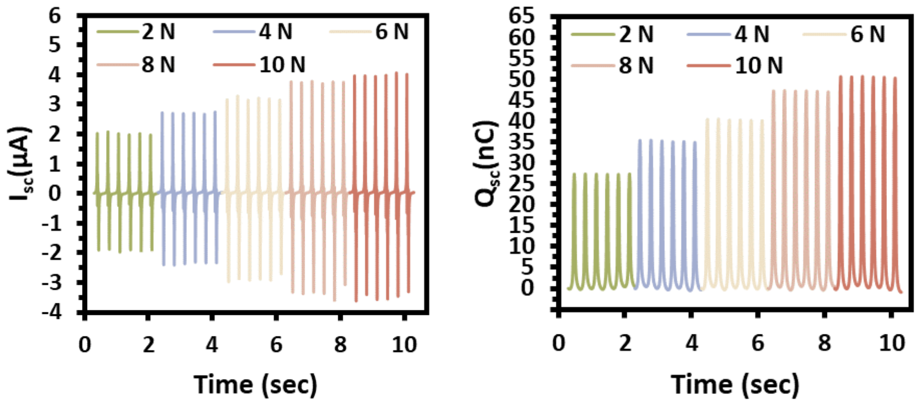

Fig. S18 I_{SC} and Q_{SC} of the SaLM-SeCNT e-textile TENG under different loading and the contact frequency is 3 Hz

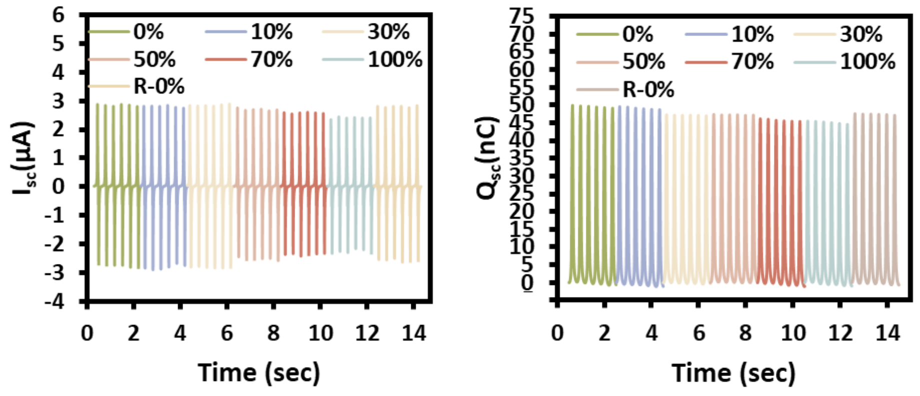

Fig. S19 I_{SC} and Q_{SC} of the SaLM-SeCNT e-textile TENG at various tensile strain levels (0-100%) and recovery states. The initial length is 20 mm, and the contact frequency is 3 Hz

Table S2 Comparison of the key characteristics of our ink based flexible TENG and the reported developments

Work	Ink	Substates	Method	Power Density	Strain	Refs.
**pnG-TENG**	Ag	Nylon	Screen printing	0.0388 W/m^2	/	[S1]
**Snow-TENG**	PEDOT: PSS	Silicon	3D printing	0.0002 W/m^2	125 %	[S2]
**TENG-FSSC**	CNT/AgNW	TPU	Screen printing	0.002 W/m^2	125 %	[S3]
**MXene-TENG**	MXene/CNF	Cotton	Immersion Coating	0.04 W/m^2	100 %	[S4]
**SPT-TENG**	Nylon-Ag-Nylon	Textile	Screen printing	0.0032 W/m^2	/	[S5]
**PPy-WSEM-TENG**	PPy	Cotton	Immersion Coating	0.000082 W/m^2	/	[S6]
**W-TENG**	Commercial ink	Cotton	Dipping	0.0066 W/m^2	/	[S7]
**Textile-TENG**	GNPs	Nylon	Drop/ Spray/ Immersion Coating	0.0308 W/m^2	/	[S8]
**PCN-TENG**	PDMS-CNTs	Silver textile	Brust Coating	0.0998 W/m^2	/	[S9]
**S-TENG**	CNTs	Kevlar fabric	Dip Coating	0.035 W/m^2	/	[S10]
**SaLM-SeCNT TENG**	SaLM/SeCNT	Multiple substrates	Writing/Screen printing	0.162 W/m^2	100%	This Work

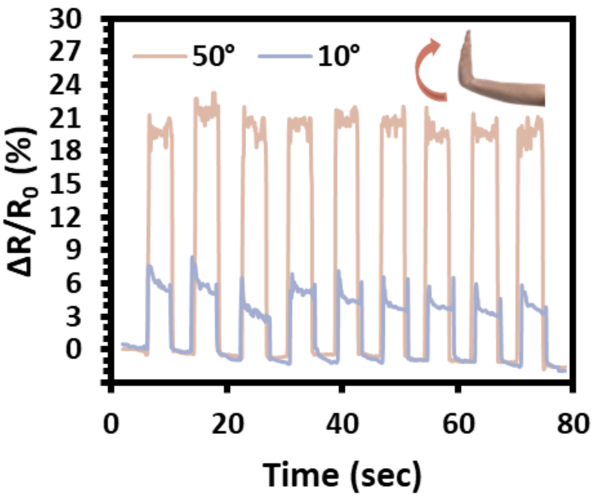

Fig. S20 Motion sensing application of the relative resistance changes of wrist bending and holding at different angles

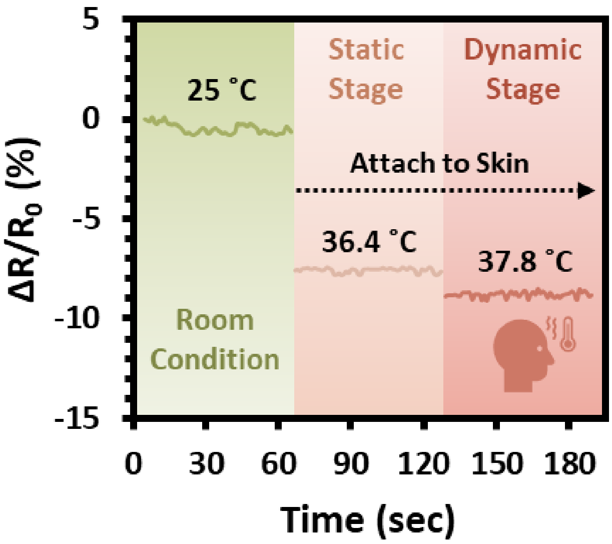

Fig. S21 Temperature sensing application of the SaLM-SeCNTs printed e-textile from room condition to human skin at static and dynamic stage (exercise)

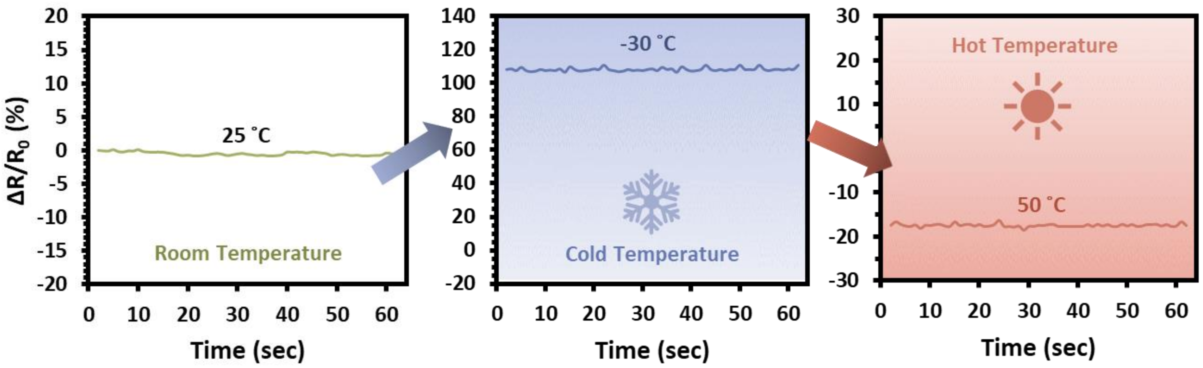

Fig. S22 Temperature sensing application of the SaLM-SeCNT printed e-textile under cool and hot conditions

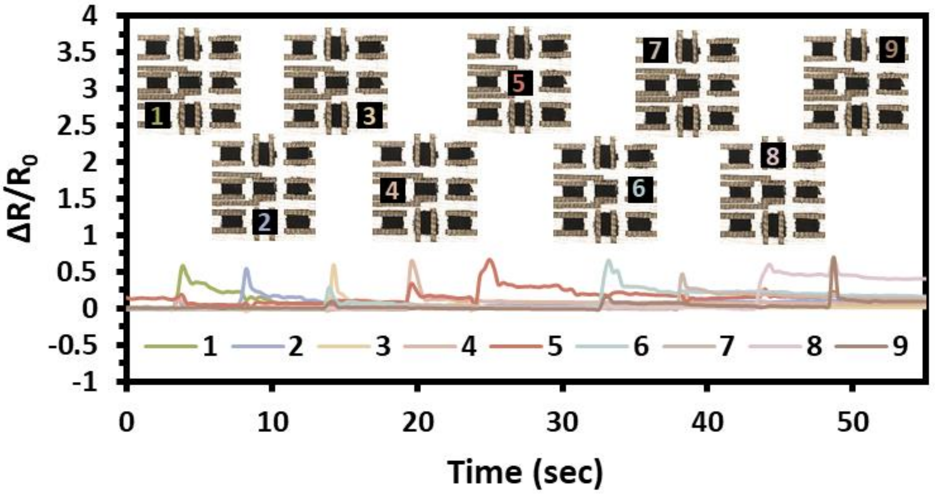

Fig. S23 Pressure sensing application of the SaLM-SeCNTs printed e-textile patterning with 9 pixels in sensing finger touch

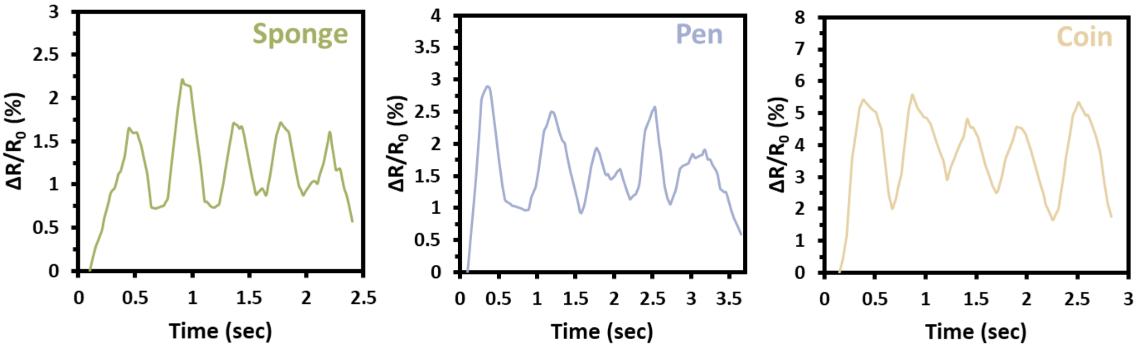

Fig. S24 Pressure sensing application of the SaLM-SeCNTs printed e-textile in sensing different objects

Supplementary References

[S1] W. Paosangthong, M. Wagih, R. Torah, S. Beeby, Textile-based triboelectric nanogenerator with alternating positive and negative freestanding grating structure. Nano Energy **66**, 104148 (2019). https://doi.org/10.1016/j.nanoen.2019.104148

[S2] A. Ahmed, I. Hassan, I.M. Mosa, E. Elsanadidy, G.S. Phadke et al., All printable snow-based triboelectric nanogenerator. Nano Energy **60**, 17–25 (2019).

https://doi.org/10.1016/j.nanoen.2019.03.032

[S3] G. Zhu, P. Ren, J. Yang, J. Hu, Z. Dai et al., Self-powered and multi-mode flexible sensing film with patterned conductive network for wireless monitoring in healthcare. Nano Energy **98**, 107327 (2022). https://doi.org/10.1016/j.nanoen.2022.107327

[S4] J. Fan, M. Yuan, L. Wang, Q. Xia, H. Zheng et al., MXene supported by cotton fabric as electrode layer of triboelectric nanogenerators for flexible sensors. Nano Energy **105**, 107973 (2023). https://doi.org/10.1016/j.nanoen.2022.107973

[S5] C. Zhang, L. Zhang, B. Bao, W. Ouyang, W. Chen et al., Customizing triboelectric nanogenerator on everyday clothes by screen-printing technology for biomechanical energy harvesting and human-interactive applications. Adv. Mater. Technol. **8,** 2201138 (2023). https://doi.org/10.1002/admt.202201138

[S6] A.R. Mule, B. Dudem, H. Patnam, S.A. Graham, J.S. Yu, Wearable single-electrode-mode triboelectric nanogenerator via conductive polymer-coated textiles for self-power electronics. ACS Sustainable Chem. Eng. **7**, 16450–16458 (2019). https://doi.org/10.1021/acssuschemeng.9b03629

[S7] Z. Zhang, J. Cai, High output triboelectric nanogenerator based on PTFE and cotton for energy harvester and human motion sensor. Curr. Appl. Phys. **22**, 1–5 (2021). https://doi.org/10.1016/j.cap.2020.11.001

[S8] I. Domingos, Z. Saadi, K.S. Sadanandan, H.A. Pocinho, D.M. Caetano et al., Printed graphene electrodes for textile-embedded triboelectric nanogenerators for biomechanical sensing. Nano Energy **115**, 108688 (2023). https://doi.org/10.1016/j.nanoen.2023.108688

[S9] P. Zhang, W. Zhang, H. Zhang, A high-performance textile-based triboelectric nanogenerator manufactured by a novel brush method for self-powered human motion pattern detector. Sustain. Energy Technol. Assess. **46**, 101290 (2021). https://doi.org/10.1016/j.seta.2021.101290

[S10] F. Yuan, S. Liu, J. Zhou, X. Fan, S. Wang et al., A smart Kevlar-based triboelectric nanogenerator with enhanced anti-impact and self-powered sensing properties. Smart Mater. Struct. **29**, 125007 (2020). https://doi.org/10.1088/1361-665x/abaf08
